# Nonaqueous Emulsion Polycondensation Enabled by a Self‐Assembled Cage‐like Surfactant

**DOI:** 10.1002/chem.202104228

**Published:** 2022-02-03

**Authors:** Sudhakar Ganta, Christoph Drechsler, Yen‐Ting Chen, Guido H. Clever

**Affiliations:** ^1^ Department of Chemistry and Chemical Biology TU Dortmund University Otto-Hahn Straße 6 44227 Dortmund Germany; ^2^ Center of Molecular Spectroscopy and Simulation of Solvent-driven Processes (ZEMOS) Ruhr-University Bochum 44801 Bochum Germany

**Keywords:** self-assembly, coordination cages, amphiphiles, emulsions, polymerization

## Abstract

Nonaqueous emulsions are crucial for a range of applications based on water‐sensitive systems such as controlled polymerizations requiring anhydrous reaction conditions and the stabilization of readily hydrolyzable reagents or pharmacologically active components. However, defined molecular surfactants to stabilize such nonaqueous emulsions are scarce. We introduce a self‐assembled coordination cage, decorated with cholesterol functionalities, to serve as a molecular surfactant for various oil‐in‐oil emulsions of immiscible organic solvents. While the positively charged cage forms the amphiphile's polar moiety, the non‐polar cholesterol appendices can bend in a common direction to stabilize the emulsion. Templated by the droplets, polycondensation reactions were carried out to produce microstructured polyurethane and polyurea materials of different particle sizes and morphologies. Further, the amphiphilic cage can encapsulate a guest molecule and the resulting host‐guest assembly was also examined as a surfactant. In addition, the aggregation behavior of the amphiphilic cage in an aqueous medium was examined.

## Graphical abstract

**A cationic self‐assembled coordination cage** was decorated with cholesterol groups attached via ethoxy chains, giving it a pronounced amphiphilic character. The nanosized cage acts as a surfactant for a variety of nonaqueous emulsions. These were employed in mini‐emulsion polymerization reactions to produce polyurethane and polyurea materials with different particle sizes and morphologies.

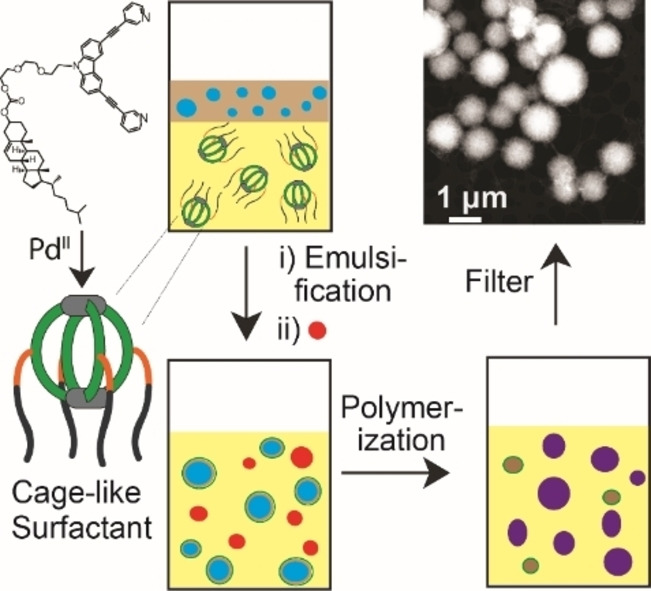

## Introduction

The self‐assembly of simple organic bridging ligands with a selection of metal ions is one of the most efficient principles to construct discrete and defined nano‐sized assemblies, varying from two‐dimensional macrocycles,[^1^, ^2^] grids,^[3]^ knots^[4]^ and helicates^[5]^ to intricate three‐dimensional cages.[^6^, ^7^, ^8^, ^9^, ^10^, ^11^, ^12^, ^13^, ^14^, ^15^, ^16^, ^17^, ^18^, ^19^] In particular, metal–organic cage structures are receiving a lot of attention because of their similarities with enzyme pockets in terms of accessible nanoconfined space and catalytic function.[^20^, ^21^, ^22^] By varying the organic ligands and choice of metal ions, numerous examples of such metal–organic cages with distinct cavity shapes and sizes were accomplished so far.[^16^, ^17^, ^23^, ^24^, ^25^, ^26^, ^27^] The defined void within these compounds is explored in molecular recognition,[^28^, ^29^] host‐guest chemistry,[^7^, ^30^, ^31^, ^32^] and supramolecular catalysis.[^33^, ^34^, ^35^, ^36^] Furthermore, certain cages were employed as functional units in rotaxanes,[^37^, ^38^] porous liquids,^[39]^ ion channels,[^40^, ^41^] liquid crystals,^[42]^ gelators,[^43^, ^44^, ^45^, ^46^] photo‐active systems,[^47^, ^48^, ^49^] and medicinal applications.[^50^, ^51^] Besides, hierarchical self‐assembly of such discrete metal–organic assemblies into higher‐order, well‐defined aggregates was also reported.[^52^, ^53^, ^54^, ^55^] Nevertheless, examples for exploiting the multifarious structures and properties of these cages as functional moieties in materials chemistry are still scarce.

Nonaqueous emulsions are formulated from two immiscible organic solvents, and are in this respect similar to conventional oil‐in‐water emulsions. However, the former type is comparatively less researched because of the difficulty of finding or designing suitable surface‐active agents.[^56^, ^57^, ^58^] Surfactants for oil‐in‐oil emulsions (o/o emulsions) must show a certain solubility in both polar and non‐polar organic solvents, but conventional surfactants usually lack this quality. To date, the majority of o/o emulsions are produced by using block copolymers[^59^, ^60^] or solid particles[^61^, ^62^, ^63^] as surfactants. The design and implementation of molecular surfactants with amphiphilic nature to emulgate immiscible organic solvents are challenging. Directed self‐assembly strategies, based on building blocks with complementary shape and reactivity, give access to functional nano structures in a modular fashion. Recently, we have shown that a defined self‐assembled cage of heteroleptic nature (carrying two sets of distinguishable ligands with different functional decoration) can yield a new type of molecular surfactant system for o/o emulsions^[64]^ which promote metal oxide microcapsule formation, as elucidated by in situ LC‐TEM.^[65]^ Here, we report an even simpler self‐assembly approach to construct a homoleptic coordination cage from one type of ligand with amphiphilic properties. The cage comprises a rather static three‐dimensional skeleton and four cholesterol moieties attached to it via triethylene glycol threads. The tetracationic cage core (polar) and the protruding cholesterol groups (non‐polar) bestow the self‐assembled architecture with pronounced amphiphilic nature. Hence, we observed that the compound can act as an efficient molecular surfactant for o/o emulsions.

O/o emulsions bear potential to realize a larger scope of water‐sensitive applications such as the stabilization of hydrolytically unstable compounds,^[66]^ serving as vehicles in drug delivery,[^67^, ^68^] finding use in material science,[^69^, ^70^] and the synthesis of polymer products.[^58^, ^71^, ^72^] In particular, emulsion polymerization is a research topic of high interest because it allows control over the synthesis of polymer‐based functional nano‐objects such as globules or capsules with well‐defined sizes and morphologies.[^58^, ^71^, ^73^, ^74^] For example, o/o emulsions were employed to yield a plethora of polyurea and polyurethane particles, which are essential materials for industrial and biological applications.[^75^, ^76^, ^77^, ^78^]

## Results and Discussion

Previously, we have introduced a banana‐shaped bis‐monodentate pyridyl ligand with carbazole backbone and studied its self‐assembly with Pd(II) to construct a lantern‐shaped [Pd_2_L_4_] coordination cage.^[79]^ Systematic investigation of this system revealed that halide ions (X=Cl^−^ or Br^−^) first template self‐dimerization of monomeric [Pd_2_L_4_]^4+^ units to yield interpenetrated double cages [3X@Pd_4_L_8_]^5+^ and excess halide ions lead to the formation of a rare triply catenated structure {*trans*‐[(PdBr_2_)_2_L_2_]}_3_. For the present study, the carbazole backbone was functionalized with a cholesterol group via a triethylene glycol linker. Replacement of the former hexyl appendix with such a significantly bulkier, hydrophobic‐hydrophilic balanced cholesterol‐triethylene glycol functionality implied a substantial variation to the solution behavior of the Pd(II)‐mediated self‐assembled product.

**Scheme 1:**
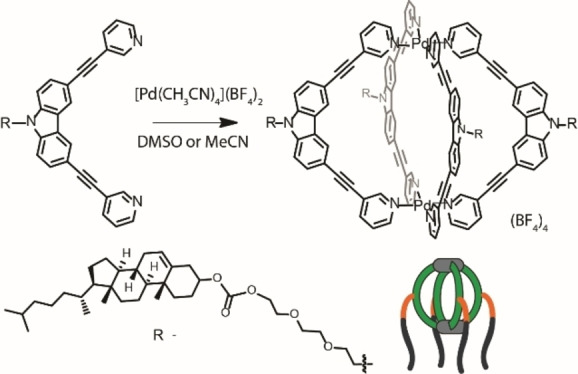
Synthesis of amphiphilic coordination cage [Pd_2_ **L**_4_]^4+^.

**Figure 1:**
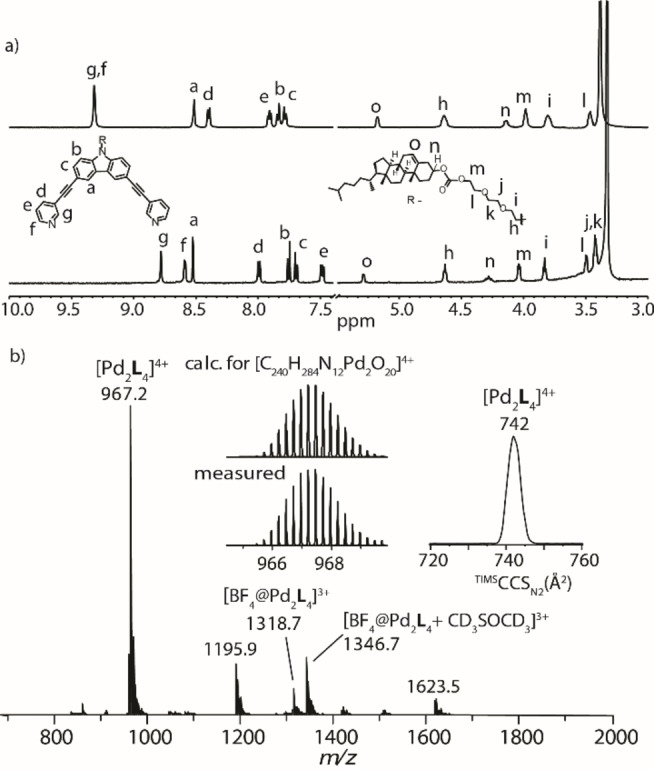
a) ^1^H NMR spectra (500 MHz, 298 K, DMSO‐*d_6_*) of ligand **L** (bottom, 2.8 mM), and cage [Pd_2_ **L**_4_]^4+^ (top, 0.7 mM); b) ESI‐MS of the cage [Pd_2_ **L**_4_]^4+^. The inset shows the ion mobility trace.

Ligand **L** was synthesized by N‐alkylation of carbazole precursor **B** with cholesteryl reagent **A** in good yield (Supporting Information, Scheme S1). The self‐assembly of this functionalized ligand **L** with Pd(II) cations at 2 : 1 ratio in DMSO‐*d*_6_ or CD_3_CN at room temperature resulted in the quantitative formation of a [Pd_2_ **L**_4_]^4+^ cage (Scheme 1). This was evidenced by ^1^H NMR spectroscopic data, HR‐ESI mass spectrometry, and DOSY studies (Figure 1, Supporting Information, Figures S10–S18). The pyridyl proton signals of ligand **L** undergo downfield shift after self‐assembly with the metal salt. A single set of signals, shifted with respect to the ligand signals, indicates the quantitative formation of a discrete coordination cage, [Pd_2_ **L**_4_]^4+^ (Figure 1a). ESI‐MS spectrometry confirmed the presence of dinuclear species with stoichiometry [Pd_2_ **L**_4_]^4+^ (Figure 1b, Supporting Information, Figure S15). Further, trapped ion mobility spectrometry (TIMS) of the [Pd_2_ **L**_4_]^4+^ ions revealed a sharp mobility signal with collisional cross‐section (CCS) value 742 Å, indicating the presence of a single species adopting a preferred conformation in the gas phase. ^1^H DOSY NMR also supported the formation of a discrete species with a hydrodynamic radius of ∼16 Å (Supporting Information, Figures S16–18). Clearly, from all these experimental results, the formation of discrete self‐assembled coordination cage product is evident. Geometry optimized models of the proposed cage structure are given in the Supporting Information, Figures S19, S21.

**Figure 2:**
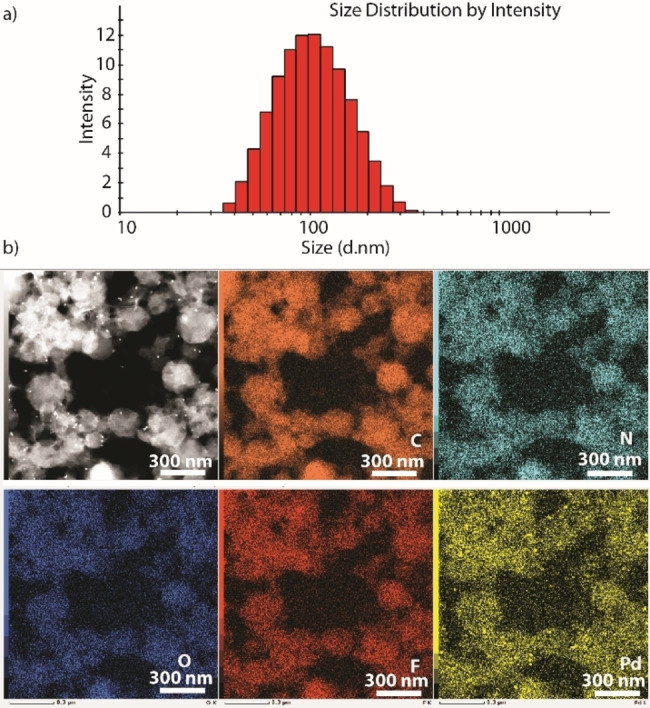
a) DLS and b) TEM analysis of the cage (0.35 mM) in MeCN: water (1 : 1) [scale bar – 300 nm; EDX analysis color code for elements – C (orange), N (cyan), O (blue), F (red) and Pd (yellow)].

Cholesterol‐based amphiphiles are well known for their ability to self‐assemble into various hierarchical nanostructures in many solvent systems.^[55]^ The presence of protruding cholesteryl attachment to the three‐dimensional cage encouraged us to study self‐aggregation of the cage in an aqueous medium. First, dynamic light scattering (DLS) was employed to check aggregation behavior. In pure MeCN, no aggregation was observed. However, in an aqueous solution (MeCN‐water, 1 : 1), the molecules aggregate giving entities with hydrodynamic diameters of about ∼50–300 nm (Figure 2a, Supporting Information, Figure S22). Next, the morphology of these aggregates was investigated using scanning transmission electron microscopy‐high‐angle annular dark‐field microscopy (STEM‐HAADF). Microscopy experiments revealed that the cage molecules self‐aggregate into spherical particles with ∼100–200 nm size (Figure 2b, Supporting Information, Figure S23). We propose that the non‐polar cholesterol skeletons stack together via hydrophobic interactions and lead to overall aggregation into spherical objects, probably via multi‐lamellar vesicle formation. Energy dispersive spectroscopy (EDS) confirms the distribution of expected elements in the observed spherical objects (Figure 2b, Supporting Information, Figure S23).

Trembling a vial containing a DMSO solution of the cage‐based amphiphile (1.4 mM, 0.2 % w/w) resulted in foam formation, which was stable over 24 h when kept aside without any interruption (Supporting Information, Figure S24). However, the same action using the ligand alone resulted in no foam formation. Thus, the amphiphilic cage molecules act as a surfactant to adsorb at the air and liquid interface to stabilize the air bubbles in oil, i. e., forming an air‐in‐oil type of emulsion. This type of nonaqueous foam where the continuous phase is an organic solvent is less explored in literature.[^72^, ^80^, ^81^]

**Figure 3:**
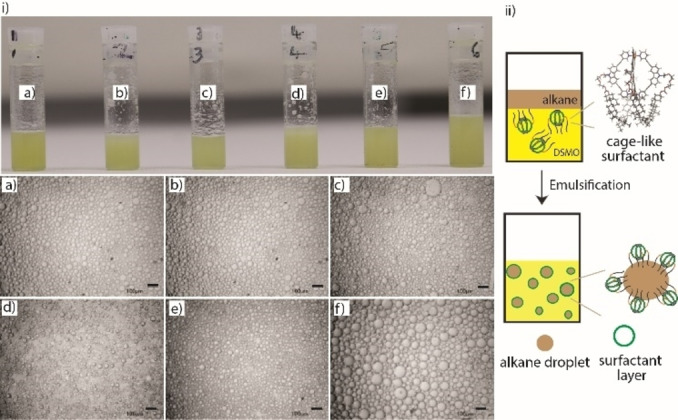
i) Top: Photographs of emulsions: a) D‐HD, b) D−O, c) D‐IPP, d) D−H, e) D−CH (a‐e are 4 : 1 w/w) and f) D‐HD (1 : 1 w/w) in the presence of cage (0.1 % w/w). Images were taken 6 h after preparation of emulsions. Bottom: Optical microscope images of corresponding emulsions (a–f), scale bar 100 μm. Images were taken 6 h after preparation of emulsions. ii) Schematic illustration of emulsion formation by the cage‐like surfactant

The formation of foam encouraged us to study the ability of the cage amphiphile to act as a surfactant for immiscible organic solvents (Supporting Information, Figure S25). First, this was examined for the DMSO‐hexadecane (D‐HD; 4 : 1) system with 0.1 % w/w cage. After 10 min of vortexing (3000 rpm), the sample turns into an opaque emulsion which is further stable over 24 h (Figure 3, and Supporting Information, Figures S26–28). Next, the disperse phase was replaced with conventional alkanes such as octane (O), hexane (H), and cyclohexane (CH), and also a fatty acid ester (isopropyl palmitate, IPP), and the result was similar (Figure 3a–e, and Supporting Information, Figures S26–28). These emulsions (D−O, D−H, D−CH, and D‐IPP) were stable for at least 24 h. In all these cases, the size of droplets of the continuous phase appeared in the range of ∼10–100 μm under an optical microscope (Figure 3). The ratio of HD to DMSO was modified from 1 : 4 to 1 : 1 without altering the surfactant percentage and also resulted in an emulsion, except the droplet size (i. e., HD) was higher (Figure 3f) compared to others. Further, several other combinations such as MeCN‐HD, DMF‐HD, CHCl_3_‐water were examined but failed to show any emulsion behavior.

We propose that the cholesterol skeletons act as a non‐polar hydrophobic tail, while the cation cage framework acts as a hydrophilic polar head, which is a typical surface‐active agent characteristic. Modeling studies suggest two alternative conformations that seem most plausible for bestowing the assembly with the required amphiphilic character. In one model, all four cholesterol‐functionalized chains bend in the same direction towards one of the cage's Pd‐faces. In this way, the overall anisotropic structure of the compound brings amphiphilicity and should allow location at the interface of two immiscible solvents. The cationic head group is associated with the more polar solvent (i. e., DMSO) while the steroid appendices interact with the non‐polar hydrocarbon solvent component (Figure 3). Alternatively, three chain extend in the same direction within the equatorial plane of the cage assembly while one extends in the opposite direction, giving the overall object the shape of a pitchfork (Supporting Information, Figures S19–21).

**Figure 4:**
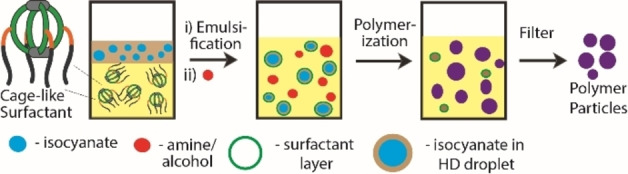
Schematic representation of oil‐in‐oil emulsion formation by the cage surfactant with different monomers in dispersed and continuous phases, followed by polymerization to yield nanostructured materials.

**Scheme 2:**
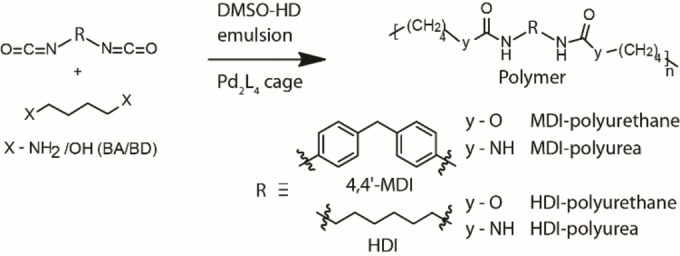
Synthesis of polyurethane and polyurea derivatives.

Emulsion polymerization is one of the best suited methods to prepare polymer nanoparticles or capsules with high molecular weight, precise morphology, and size.^[73]^ Polyurethanes (PUT) and polyurea (PU) are widely used polymer materials in coatings, adhesives, medical science, manufacturing, and construction industries.[^75^, ^82^, ^83^] These polymers are produced by condensation of di/poly‐isocyanates with di/poly‐alcohols/amines in suitable conditions. Efforts to prepare PUT and PU nanoparticles/capsules via different mini‐emulsion polymerization techniques were reported.[^76^, ^77^, ^78^, ^84^, ^85^] In this context, aqueous emulsions were also used to produce nanoparticles/capsules of polyurethanes and polyurea. However, owing to the violent reactivity of isocyanates with water, nonaqueous emulsions are preferred to accomplish the controlled polymerization of isocyanates. Here, emulsion polycondensation reactions were conducted by employing o/o emulsions of D‐HD (DMSO‐hexadecane, 4 : 1) with 0.2 % cage surfactant (Figure 4, section 8 in Supporting Information). 4,4’‐Diphenylmethane diisocyanate (4,4’‐MDI) and hexamethylene diisocyanate (HDI) were chosen as isocyanate monomers, which were reacted with an equimolar amount of 1,4‐butanediol (BD)/1,4‐diaminobutane (BA) monomers in the emulsion polymerization technique to yield PUT and PU, respectively (Scheme 2). PUTs were synthesized by employing dibutyltin dilaurate (DBTDL) catalyst (0.1 % equivalent) at 60 °C for 4 h, while PU was produced at room temperature for 1 h without any catalyst. Isocyanate monomers and DBTDL were soluble in HD, while BA/BD monomers and the cage surfactant were freely soluble in DMSO. Thus, mini‐emulsion polymerization was performed at/in the droplets of the dispersed phase containing the isocyanate monomer, which reacts with the other monomer (i. e., either amine or alcohol) presented in the continuous phase.

**Figure 5:**
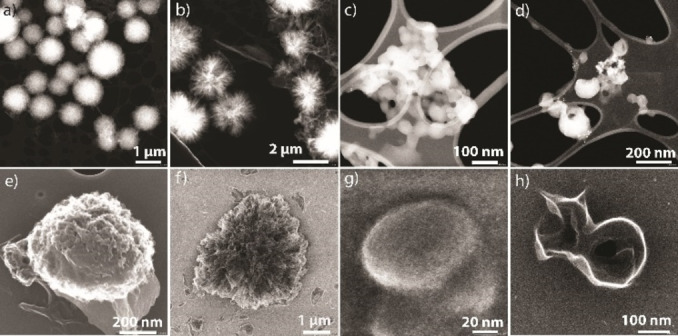
STEM‐HAADF (a–d) and SEM (e‐f) images of 4,4’‐MDI‐PU (a and e), HDI‐PU (b and f), 4,4’‐MDI‐PUT (c and g), and HDI‐PUT (d and h).

Next, we employed this mini‐emulsion polycondensation technique to prepare PU. Amines react quickly with isocyanates, unlike alcohols at room temperature, even in the absence of a catalyst. First, isocyanate monomer (1 equivalent) was pre‐dissolved in HD solvent, then introduced into DMSO containing 0.2 % cage surfactant to prepare the mini‐emulsion by vortexing at 3000 rpm for 10 min. The other monomer, BA (1 equivalent), was introduced into the colloidal emulsion solution and continued stirring for one hour. Polymer nanoparticles were isolated by adding excess water, filtration, followed by washing with hexane. The morphology of the nanoparticles was examined by TEM. Interestingly, PU obtained from 4,4’‐MDI, and HDI isocyanate monomers differ in their overall structure (Figure 5 and Supporting Information, Figures S29, S30). Spherical polymeric balls with ∼500–2000 nm size were observed for 4,4’‐MDI‐PU. The outer surface of the polymer balls is very rough and porous (Figure 5a, 5e and Supporting Information, Figure S29).

Whereas the morphology of HDI‐PU looks like asymmetrical cotton wool balls (∼2–3 μm size) with fibrous threads as shown in Figure 5b and Supporting Information Figure S30. This observation suggested that diffusion of the amine compound into the isocyanates occurs while polycondensation is subjective to the isocyanates property.

Next, we employed this mini‐emulsion polycondensation technique to prepare PUT polymers. PUT nanomaterials were also designed similarly to PU. Initially, isocyanate monomer (4,4’‐MDI/ HDI) and DBTDL (catalyst) were pre‐dissolved in HD solvent. Then emulsion with mini droplets of disperse phase (HD with the isocyanate monomer and catalyst) was created in the continuous phase, i. e., DMSO (containing 0.2 % w/w cage surfactant). After emulsification, the equimolar amount of another monomer, BD (which is freely soluble in DMSO), was introduced and stirred for 15 minutes at ambient temperature to ensure effective distribution of BD in the emulsion. Then the temperature was raised abruptly to 60 °C thus allowing polycondensation of monomers within the droplets of the mini‐emulsion. After four hours, excess water was added to the reaction and polymer nanoparticles were isolated by filtration, followed by washing with hexane. Morphological characteristics of the particles were investigated by TEM. TEM images indicated aggregated particles for 4,4’‐MDI and HDI PUTs (Figure 5c, 5d and Supporting Information, Figures S31, S32). A careful inspection of these aggregates suggested that a number of pearl‐shaped particles with less than 100 nm size is conglomerated with neighboring particles. Probably, high reaction temperatures compared to the former experiments cause these changes in the emulsion behavior, thus polymer morphology. Further, the polymers were characterized by infrared spectroscopy (IR). The IR spectra of given polymer particles of 4,4’‐MDI and HDI exhibited characteristic bands of urea or urethane (particularly ‐C=O‐stretching vibrations in the region of 1700–1600 cm^−1^, Supporting Information, Figure S33, S34).

**Figure 6:**
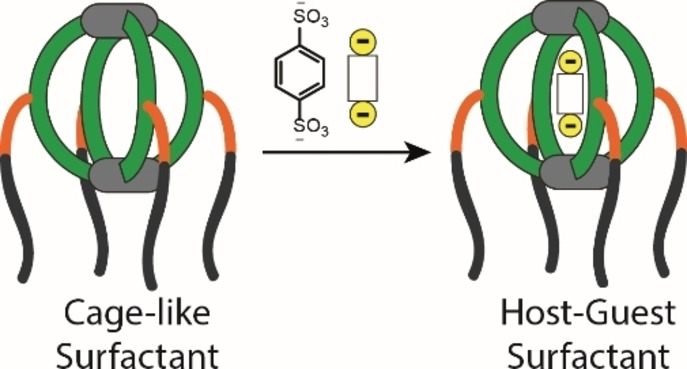
Formation of host‐guest assembly [**G**@Pd_2_ **L**_4_]^2+^.

Coordination cages with defined cavities are known for their guest encapsulation abilities.^[86]^ Since the amphiphilic cage possesses a suitably sized inner space, we anticipated guest molecules such as benzene 1,4 disulphonate (**G**) to be bound (Figure 6). Indeed, the addition of one equivalent **G** to a solution of [Pd_2_ **L**_4_]^4+^ in DMSO‐*d_6_* resulted in formation of host‐guest assembly [**G**@Pd_2_ **L**_4_]^2+^, confirmed by ^1^H NMR spectroscopy and HR‐ESI‐MS (Supporting Information, Figures S35, S36). In the ^1^H NMR spectrum of [**G**@Pd_2_ **L**_4_]^2+^, signals of the inward pointing protons (i. e. H_g_ and H_a_) were broadened and downfield shifted by about 0.32 and 0.10 ppm compared to the free cage, indicating formation of the host‐guest inclusion complex. The ESI‐MS spectrum also supported the existence of a host‐guest assembly by showing a prominent signal at *m/z*=2052.9 that corresponds to [**G**@Pd_2_ **L**_4_]^2+^.

We then compared the emulsification ability of the host‐guest assembly with that of the free host. [**G**@Pd_2_ **L**_4_]^2+^ was able to emulsify the D‐HD (4 : 1) system (0.1 % w/w), and the droplet size of the dispersed phase (HD) was found to be <100 μm (Supporting Information, Figure S37), which is comparable to results obtained in the free cage emulsification studies. Hence, inclusion of the guest inside the cavity did not significantly alter the amphiphilic nature of the cage. Next, also the emulsion templated polycondensation giving 4,4’‐MDI‐PU particles was studied using [**G**@Pd_2_ **L**_4_]^2+^ instead of the free cage. Electron microscopy analysis revealed polyurea particles of similar dimensions as with the free cage surfactant, but with a more inhomogeneous, less well‐defined spherical morphology, which might be a result of the reduction of the net charge of the amphiphile's polar head group (Supporting Information, Figure S38).

## Conclusion

In summary, a new supramolecular coordination cage with external functionalization is reported. The self‐assembled cage was characterized by NMR, ESI‐MS, and its structure was proposed with the help of geometry optimization studies, based on the known X‐ray structure of the parental lantern‐shaped [Pd_2_L_4_] cage. The hydrophobic steroid skeleton and cationic cage framework along with the ethoxy groups, import anisotropic nature to the cage. Thus, the overall molecule behaves as an amphiphile. In an aqueous solution, the amphiphilic cages aggregate to form spherical particles, which were visualized by TEM and DLS. The amphiphilic cage molecule also acts as a surface‐active agent for o/o type emulsions such as DMSO‐hexadecane. Moreover, the amphiphilic cage was shown to retain its guest binding ability and a host‐guest adduct was demonstrated to have an emulsification ability similar to the free cage.

Further, the compounds were employed in the mini‐emulsion polymerization technique to produce polyurethane and polyurea materials with different sizes and shapes. The herein reported results highlight the scope of coordination cage functionalization and their potential for application in materials chemistry. The modular self‐assembly approach to yield amphiphilic coordination cages and host‐guest assemblies may promote the systematic development of new supramolecular surfactants for oil‐in‐oil emulsions, thereby opening the window for their implementation in pharmaceutical, medicinal, and material chemistry. Further, the homogeneous nature of the emulsion reaction medium provides opportunities for continuous reactor polymerization, with control over shape and size of desired polymer products.

## Experimental Section

The detailed synthesis and characterization of all the compounds, DLS, emulsification experiments, calculation details and TEM methods are described in the Supporting Information.

## Conflict of interest

The authors declare no conflict of interest.

1

## Supporting information

As a service to our authors and readers, this journal provides supporting information supplied by the authors. Such materials are peer reviewed and may be re‐organized for online delivery, but are not copy‐edited or typeset. Technical support issues arising from supporting information (other than missing files) should be addressed to the authors.

Supporting Information

## Data Availability

The data that support the findings of this study are available from the corresponding author upon reasonable request.
