# Supplementary material for: Nonaqueous Emulsion Polycondensation Enabled by a Self‐Assembled Cage‐like Surfactant
Source: Chemistry. 2022 Feb 3;28(12):e202104228. doi: 10.1002/chem.202104228 (PMC9303455; doi:10.1002/chem.202104228)
Supplement: Supplementary file 1 — Supporting Information [file CHEM-28-0-s001.pdf]

Chemistry–A European Journal

Supporting Information

Nonaqueous Emulsion Polycondensation Enabled by a Self-Assembled Cage-like Surfactant

Sudhakar Ganta, Christoph Drechsler, Yen-Ting Chen, and Guido H. Clever*

Contents:

1) Experimental section ... 2
1.1 Materials and methods ... 2
1.2 Experimental Procedures ... 2
2) Synthesis ... 3
2.1 Precursor A ... 3
2.2 Precursor B ... 4
2.3 Ligand L ... 5
2.4 Cage Synthesis ... 10
3) ^{1}H-DOSY spectroscopy ... 14
4) Geometry optimization of cage $[Pd_2L_4]^{4+}$ ... 16
5) Hierarchical assembly of aggregates based on the amphiphilic cage ... 19
5.1 DLS analysis of cage in MeCN: Water (1:1) ... 19
5.2 TEM analysis of cage in MeCN: Water (1:1) ... 19
6) Foam formation ... 20
7) Oil-in-oil emulsification ... 20
7.1 Method ... 20
8) Polymerization ... 22
8.1 Synthesis of polyurea nanoparticles ... 22
8.2 Synthesis of polyurethane nanoparticles ... 22
8.3 FT-IR spectra of polyurethane particles ... 25
9) Host-guest chemistry of the amphiphilic cage ... 27
9.1 ^{1}H NMR spectroscopy studies of the host guest assembly: titration of G to $[Pd_2L_4]^{4+}$cage 27
9.2 ESI-MS of the $[G@Pd_2L_4]^{2+}$ ... 28
9.3 Oil-in-oil emulsification by host-guest adduct $[G@Pd_2L_4]^{2+}$. ... 28
9.4 Synthesis of polyurea nanoparticles by employing $[G@Pd_2L_4]^{2+}$ as surfactant. ... 28
10) References ... 29

1) Experimental section

1.1 Materials and methods

Unless otherwise stated, all chemicals were obtained from commercial sources and used as received. NMR spectroscopic data was measured on the spectrometers Bruker AV 500 Avance NEO and AV 600 Avance III HD. For ^{1}H chemical shifts were calibrated to the solvent lock signal. Proton signals were assigned with the aid of 2D NMR spectra. ^{1}H DOSY NMR spectra were recorded with a dstebpgp3s pulse sequence with diffusion delays D20 of 0.06-0.10 s and gradient powers P30 of 800 to 2000 µs. Electrospray ionization (ESI) mass spectra were recorded on a Bruker timsTOF Mass Spectrometer. Fourier transform infrared spectrometer (FT-IR) data were measured on Perkin Elmer Spectrum Two. Dynamic light scattering experiments were performed on a Malvern Zetasizer ZS nano instrument, with a single 173° scattering angle, and at 25 °C temperature. STEM measurements were done using a JEOL JEM-2800 with Schottky field emission cathode operated at 200 kV. A Gatan OneView IS camera (4K x 4K with 25fps) is equipped for conventional CTEM images. Also, HAADF, BF, SEM detectors are equipped for STEM images. The collection angle of BF and HAADF are 28 mrad and 34-157 mrad, respectively. Dual SDD X-ray detectors are used to capture EDS signals for elemental mapping with solid angle of 0.95 srad, and with 133 eV of spectral resolution. The CTEM resolution is 0.2 nm with information limit of 0.09 nm. The STEM resolution is 0.19 nm, and SEM resolution is 0.43 nm. The spherical aberration (Cs) is 0.7mm, and chromatic aberration (Cc) is 1.3 mm in the equipment. Optical microscopy images were captured using KEYENCE VH - Z100UR digital microscope. Geometry optimized models of structures were constructed using Wavefunction SPARTAN′18 and first optimized on semiempiric PM6 level of theory without constraints.

1.2 Experimental Procedures

Where necessary, experiments were performed under nitrogen atmosphere using standard Schlenk techniques. Chemicals and standard solvents were purchased from Sigma Aldrich, Acros Organics, Carl Roth, TCI Europe, VWR, ABCR and used as received, if not mentioned differently. Dry solvents were purchased or purified and dried over absorbent-filled columns on a GS-Systems solvent purification system (SPS). Reactions were monitored with thin layer chromatography (TLC) using silica coated aluminum plates (Merck, silica 60, fluorescence indicator F254, thickness 0.25 mm). For column chromatography, silica (Merck, silica 60, 0.02–0.063 mesh ASTM) was used as the stationary phase.

2) Synthesis

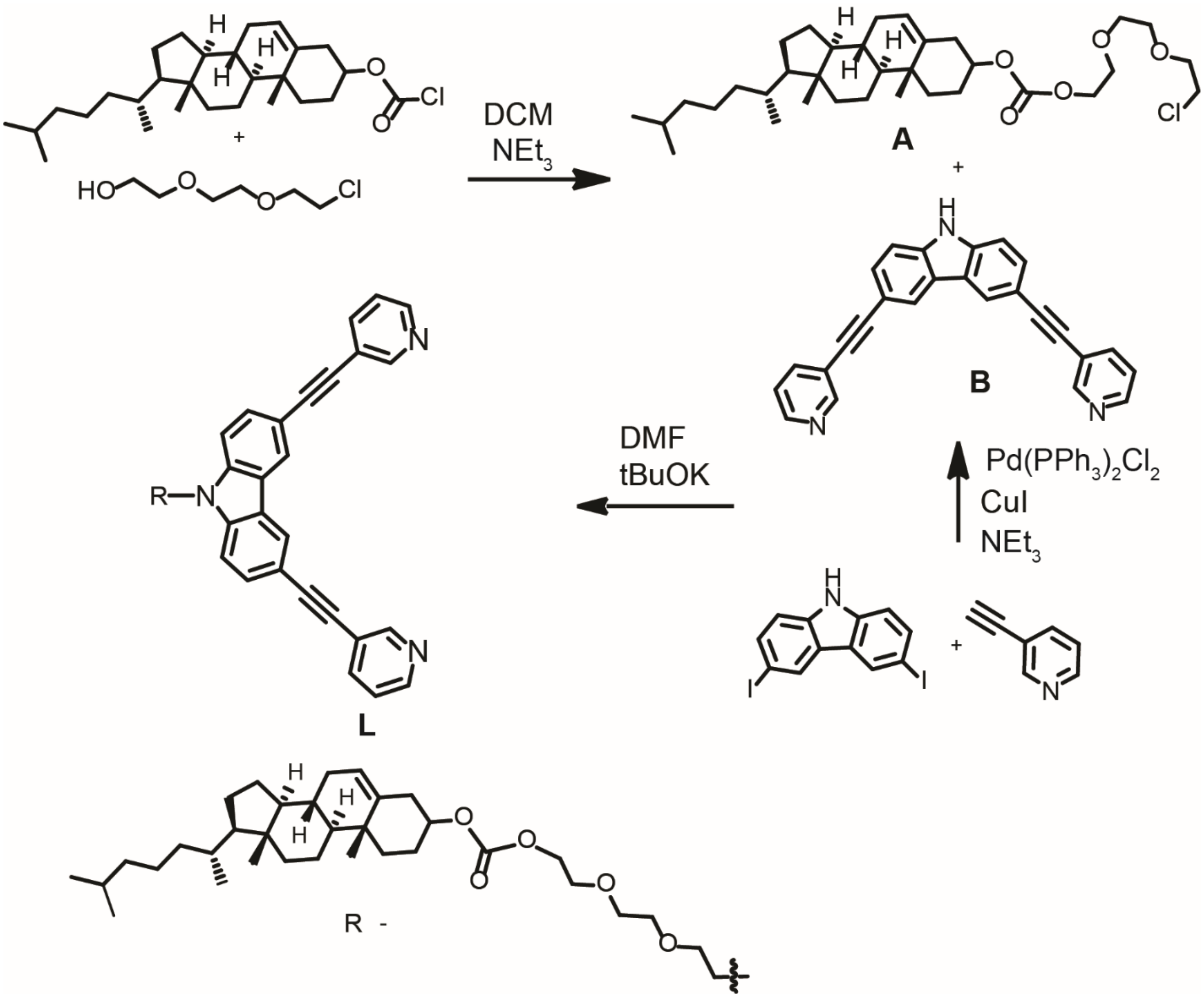

Scheme S1. Synthesis of precursors **A**, **B** and ligand **L**.

2.1 Precursor A

A mixture of cholesteryl chloride (800 mg, 1.78 mmol, 1 eq.), and 2-[2-(2-chlorethoxy)-ethoxy]-ethanol (335 mg, 1.78 mmol, 1.0 eq.) were dissolved in 10 mL dry dichloromethane (DCM). Then triethylamine (1 mL) was added and stirred for 18 h at room temperature. DCM (10 mL), water (5 mL) was added to the mixture, and the organic phase was washed with water (3 x 5 mL), dried over $MgSO_4$, filtrated. A gummy white solid (900 mg, yield 87 %) was obtained after evaporation of the solvent under reduced pressure. The product was used without further purification.

^{1}H NMR (500 MHz, $CDCl_3$): δ [ppm] = 5.38 (d, J = 5.5 Hz, 1H), 4.47 (q, J = 5.5 Hz,1H), 4.28-4.27 (m, 2H), 3.77-3.62 (m, 10H), 2.44-2.36 (m, 2H), 2.12-0.87 (m, 38H), 0.68 (s, 3H).

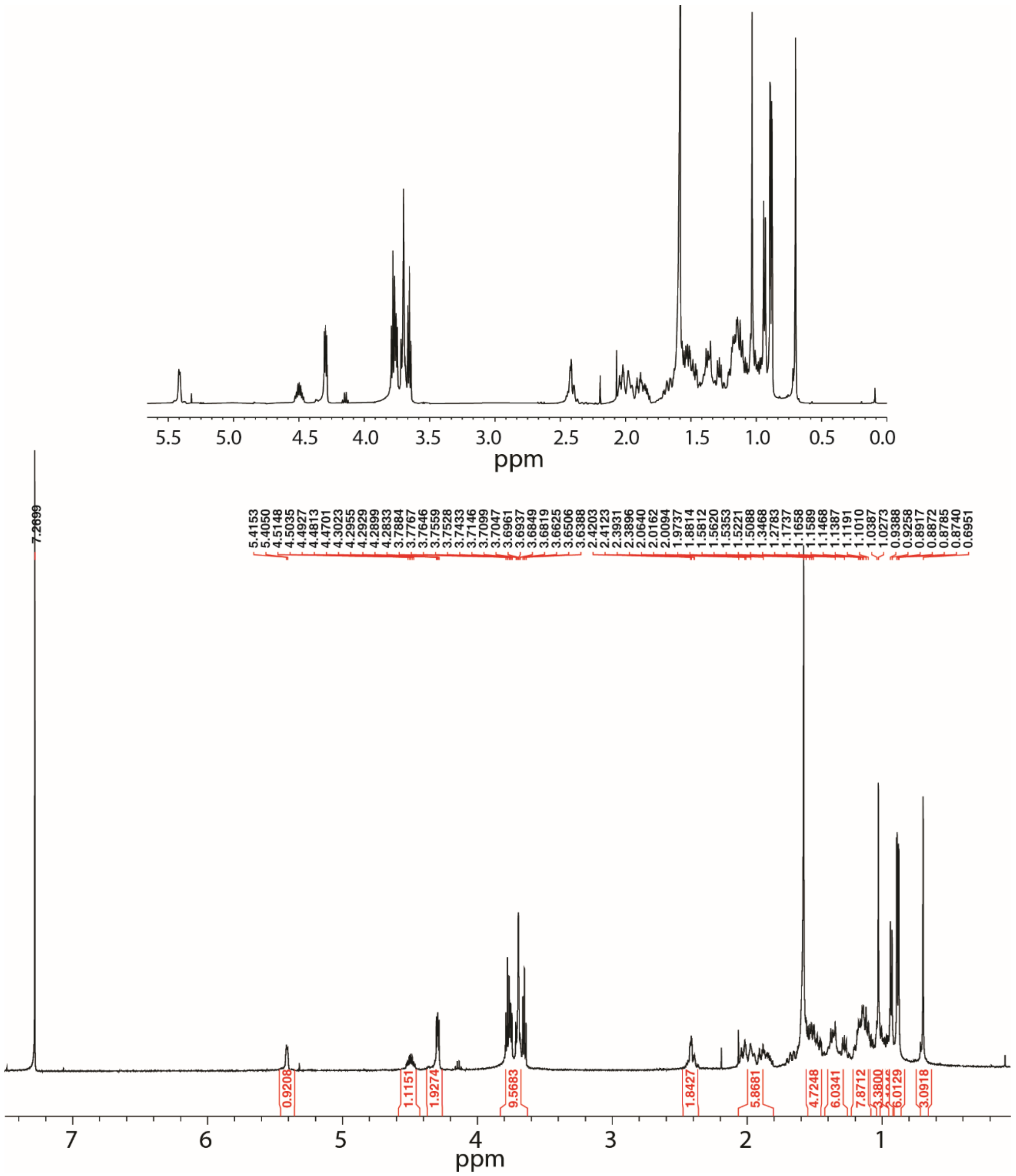

Figure S1: ^{1}H NMR spectrum (500 MHz, 298K, $CDCl_3$) of **A**: Full spectrum (bottom), selected region (top).

ESI-FTICR-HRMS [$C_{34}H_{57}ClO_5$ + HCOOH + H]$^+$: found: 624.46; calc.: 624.38

2.2 Precursor B

A mixture of 3,6-diiodo-9H-carbazole (1000 mg, 24 mmol, 1 eq.), 3-ethynylpyridine (703 mg, 71 mmol, 3 eq.) and CuI (45 mg, 3 mm, 0.13 eq.) were dissolved in triethylamine (20 mL) then degassed for 30 min. Then $Pd(PPh_3)_2Cl_2$ (168 mg, 3 mm, 0.13 eq.) was added to the above solution and stirred for 18 h at 60 °C. Then the reaction mixture was cooled down to room temperature and filtered. The precipitate was washed with aqueous ammonia (10 mL) followed by ethyl acetate (2*25 mL) to give a pale-yellow solid (750 mg, yield 85%). The pale-yellow solid was used without further purification.

^{1}H NMR (500 MHz, DMSO-d_6): δ [ppm] = 11.86 (s, 1H), 8.79 (s, 2H), 8.63-8.58 (b, 2H), 8.51 (s, 2H), 8.00 (dt, J = 7.8 Hz, 2H), 7.65 (dd, J = 6.9 Hz, 2H), 7.59 (d, J = 8.4 Hz, 2H), 7.49 (dd, J = 7.8 Hz, 2H).

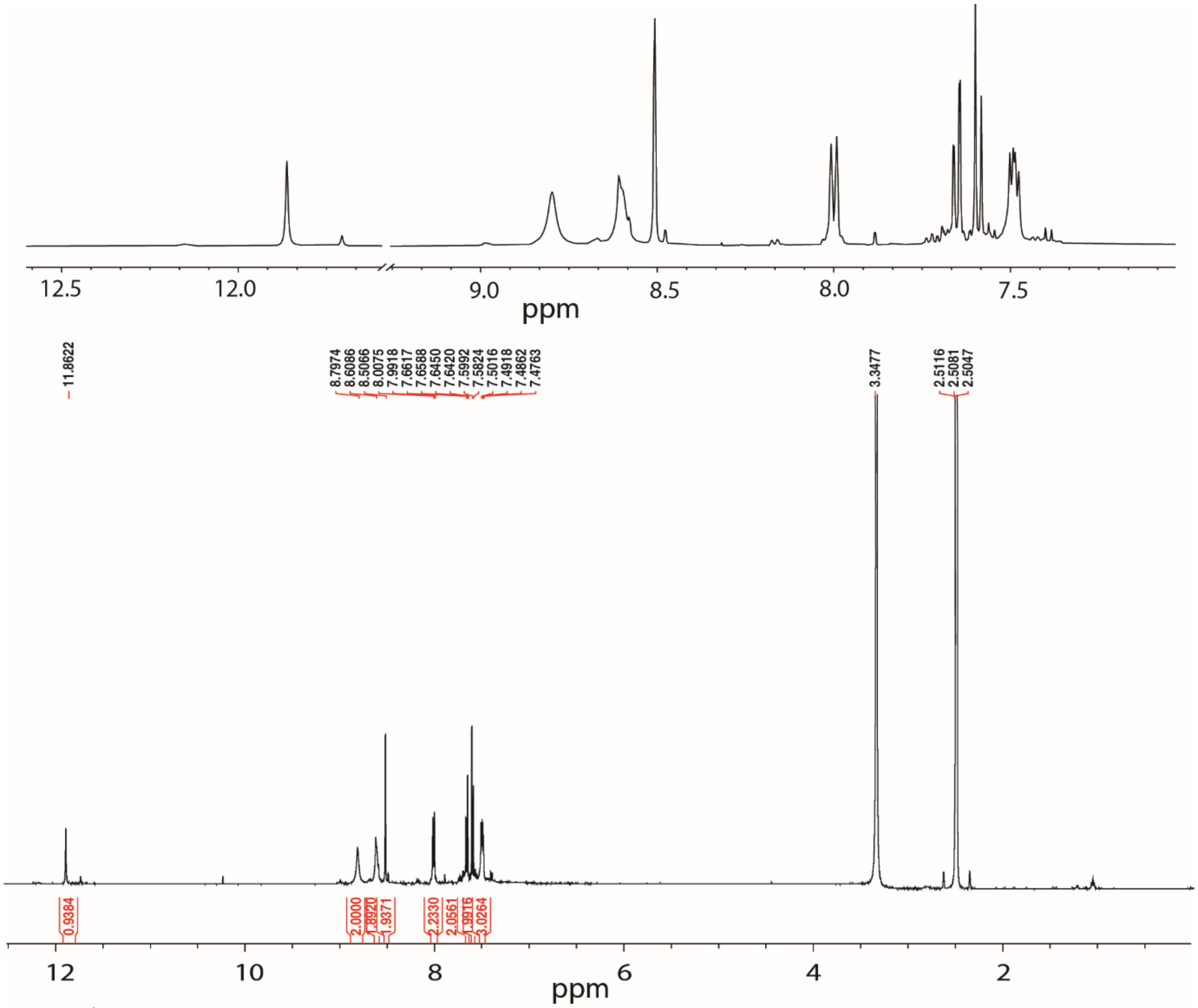

Figure S2: ^{1}H NMR spectrum (500 MHz, 298K, DMSO-d_6) of **B**: Full spectrum (bottom), selected region (top).

2.3 Ligand L

A mixture of precursor **A** (330 mg, 0.57 mmol, 1.5 eq.) and precursor **B** (140 mg, 0.38 mmol, 1 eq.) were dissolved in a 5 mL dry DMF solvent containing K_2CO_3 (108 mg, 0.8 mmol, 2 eq.) and KI (10 mg, 0.06 mmol, 0.15 eq.). The mixture was degassed and stirred for 48 h at 80 °C, then cooled to rt. Ethyl acetate (25 mL), water (10 mL) was added to the mixture, and the organic phase was washed with water (3 x 10 mL), dried over $MgSO_4$, filtrated, and the solvent was evaporated under reduced pressure. The crude residue was purified by flash chromatography on silica gel (Pentane: Ethyl acetate = 9: 1 to 1:1), followed by GPC to give the product as a yellow solid (260 mg, 0.28 mmol, 75 %).

^{1}H NMR (600 MHz, $CDCl_3$): δ [ppm] = 8.81 (d, J = 1.5 Hz, 2 H), 8.55 (dd, J = 4.8 Hz, 2 H), 8.29 (d, J = 1.0 Hz, 2 H), 7.86 (d, J = 1.0 Hz, 2 H), 7.86 (dt, J = 7.8 Hz, 2 H), 7.67 (d, J = 8.4 Hz, 2 H), 7.31 (dd, J = 7.8 Hz, 2 H), 5.36 (d, J = 5.5 Hz, 1H), 4.52 (t, J = 5.6 Hz, 2H), 4.45 (q, J = 5.5 Hz, 1H), 4.17 (t, J = 4.8 Hz, 2H), 3.90 (t, J = 5.8 Hz, 2H), 3.56-3.50 (m, 6H), 3.80-3.63 (m, 10H), 2.40–2.32 (m, 2H), 1.97–0.85 (m, 38H), 0.67 (s, 3H).

^{1}H NMR (500 MHz, DMSO-*d*$_6$): δ [ppm] = 8.83 (dd, *J* = 1.1 Hz, 2H), 8.64 (dd, *J* = 4.8 Hz, 2H), 8.57 (d, *J* = 1.2 Hz, 2H), 8.04 (dt, *J* = 7.8 Hz, 2H), 7.81 (d, *J* = 8.5 Hz, 2H), 7.74 (dd, *J* = 8.2 Hz, 2H), 7.54 (d, *J* = 7.9 Hz, 2H), 5.34-5.33 (b, 1H), 4.68 (t, *J* = 4.76 Hz, 2H), 4.33 (q, *J* = 3.8 Hz, 1H), 4.17 (t, *J* = 4.8 Hz, 2H), 4.09 (m, 2H), 3.88 (t, *J* = 5.5 Hz, 2H), 3.49-3.46 (m, 4H), 2.36–2.29 (m, 2H), 1.98–0.89 (m, 38H), 0.62 (s, 3H).

^{13}C NMR (150 MHz, $CDCl_3$): δ [ppm] = 154.5, 152.2, 148.2, 140.9, 139.3, 138.3, 129.9, 124.3, 123.1, 123.0, 122.5, 120.1, 113.5, 109.6, 93.7, 84.6, 77.9, 71.0, 70.7, 69.4, 69.0, 66.6, 56.7, 56.1, 49.9, 43.6, 42.3, 39.7, 39.5, 38.0, 36.8, 36.5, 36.2, 35.8, 31.9, 31.8, 28.2, 28.0, 27.6, 24.3, 23.8, 22.8, 22.6, 21.0, 19.2, 18.7, 11.9.

ESI-FTICR-HRMS [$C_{60}H_{71}N_3O_5$ - H]$^+$: found: 912.5307; calc.: 912.5310

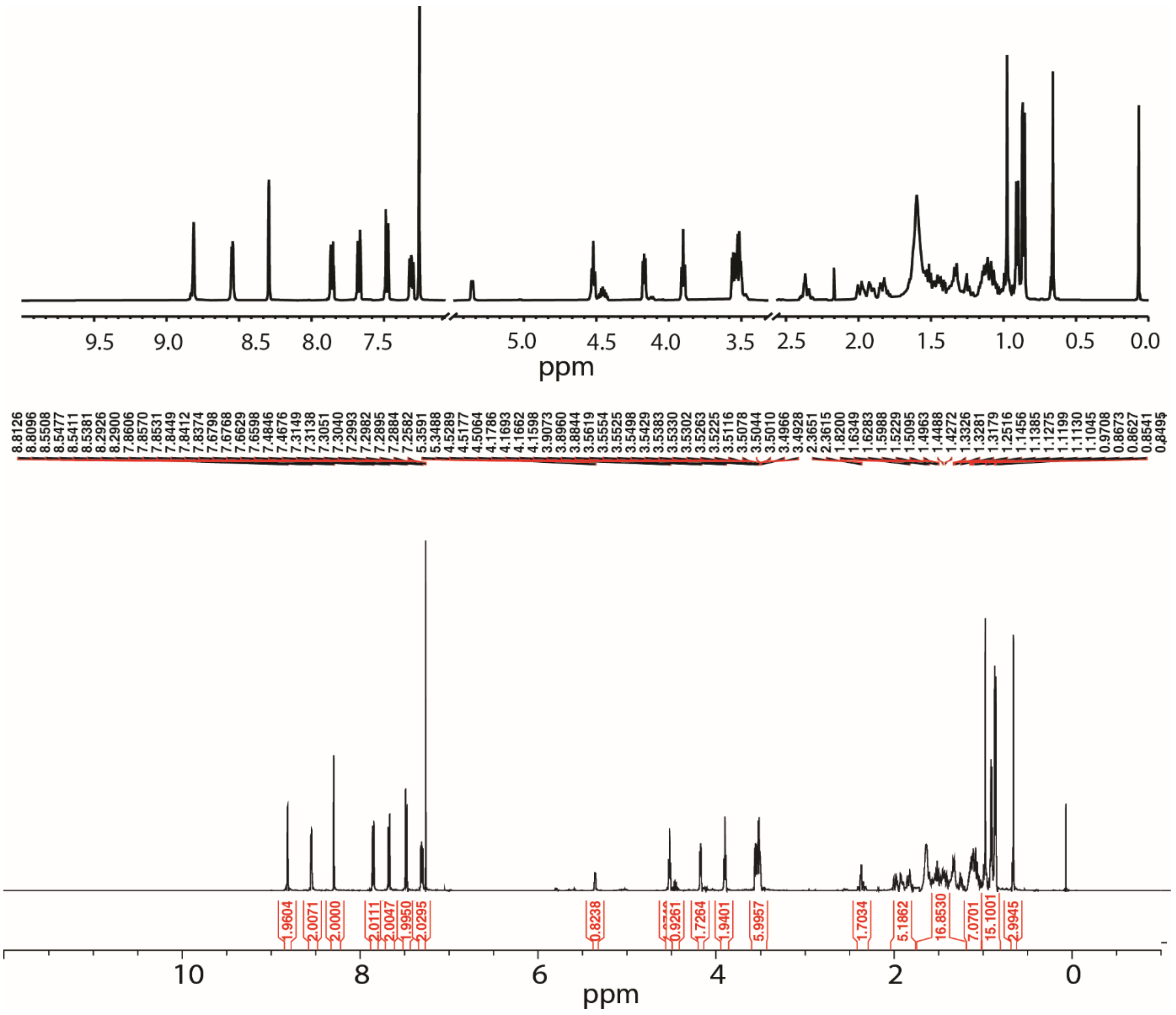

Figure S3: ^{1}H NMR spectrum (600 MHz, 298K, $CDCl_3$) of ligand **L**: Full spectrum (bottom), selected region (top).

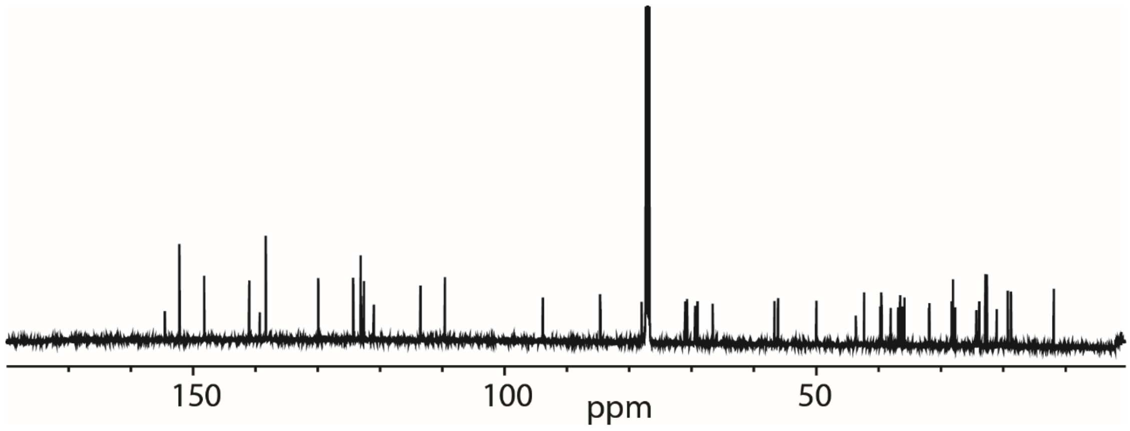

Figure S4: ^{13}C NMR spectrum (150 MHz, 298K, $CDCl_3$) of ligand **L**

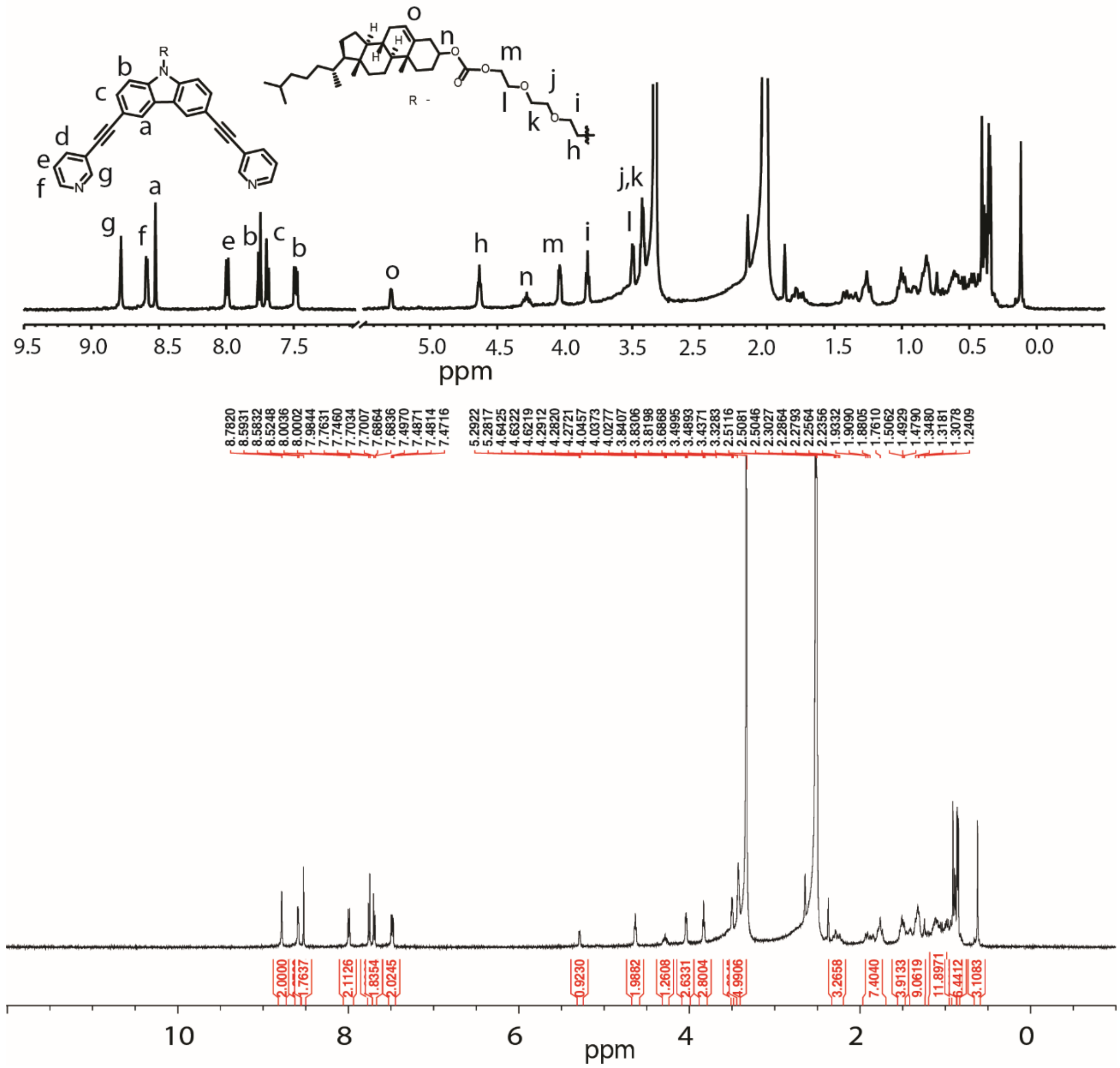

Figure S5: ^{1}H NMR spectrum (500 MHz, 298K, DMSO-d_6) of ligand **L**: Full spectrum (bottom), selected region (top).

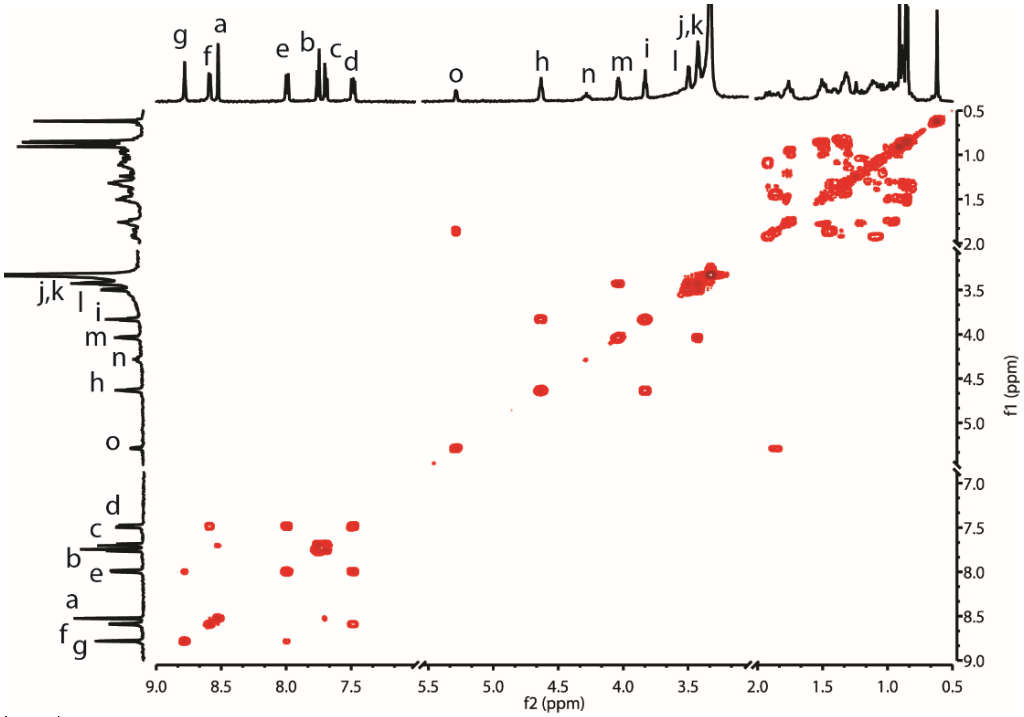

Figure S6: ^{1}H - ^{1}H COSY NMR spectrum (500 MHz, 298K, DMSO-d_6) of ligand **L**

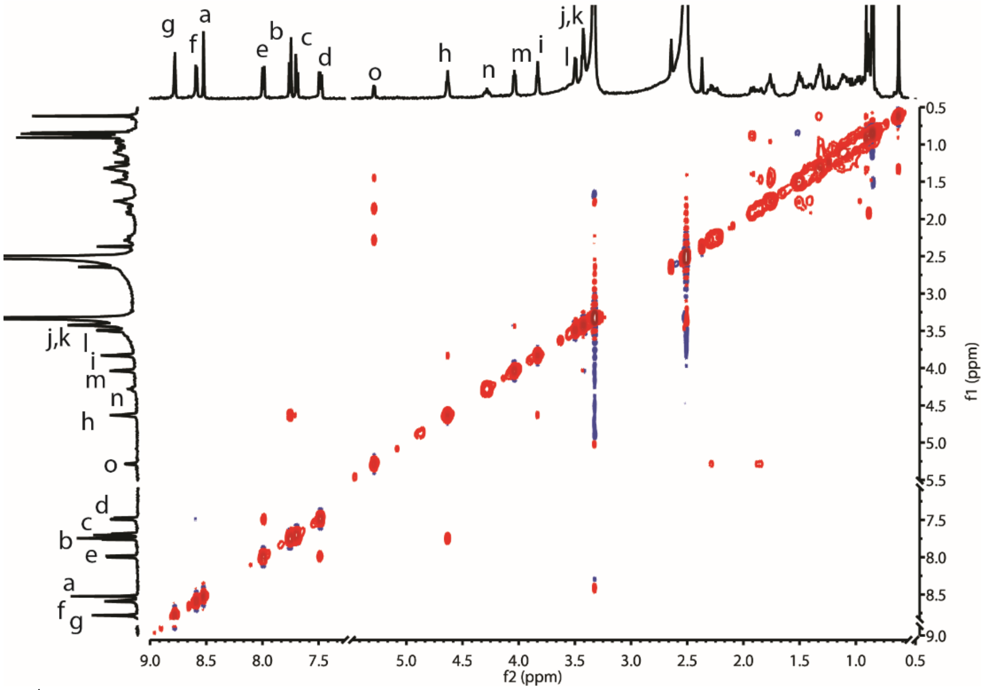

Figure S7: ^{1}H - ^{1}H NOESY NMR spectrum (500 MHz, 298K, DMSO-d_6) of ligand **L**

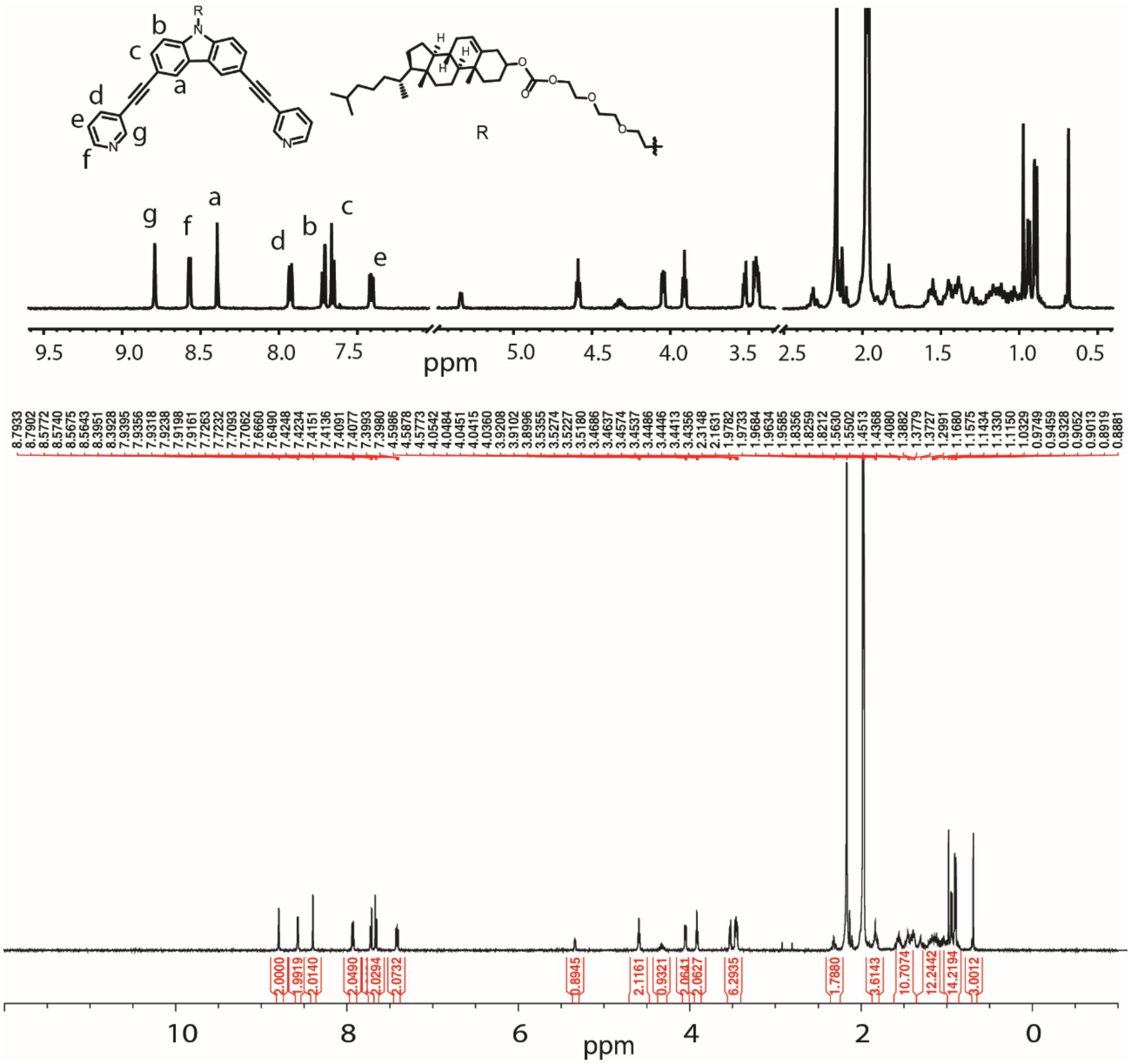

Figure S8: ¹H NMR spectrum (500 MHz, 298K, CD_3CN) of ligand **L**: Full spectrum (bottom), selected region (top).

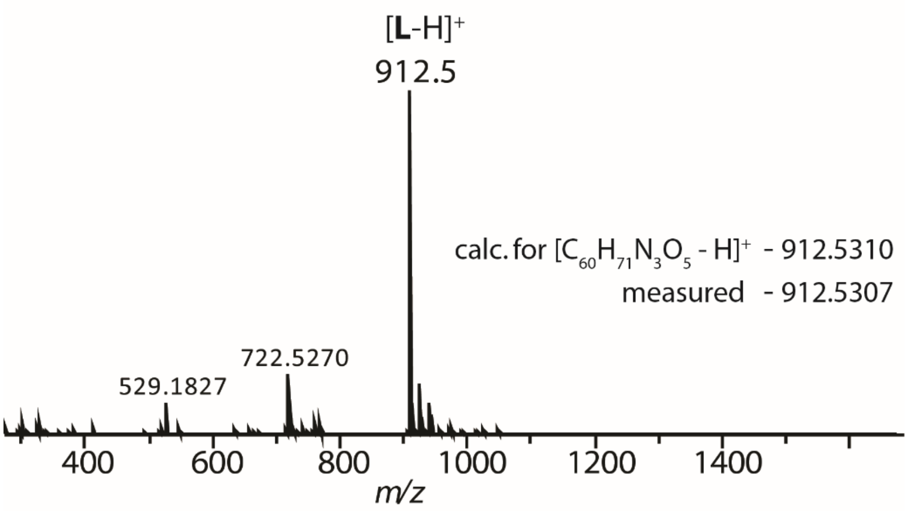

Figure S9: ESI-MS of the ligand **L**

2.4 Cage Synthesis

Ligand **L** (1.27 mg, 1.4 µmol) in 425 µL of DMSO-d_6 or CD_3CN was mixed with $[Pd(CH_3CN)_4](BF_4)_2$ (0.75 µmol, 75 µL of a 10 mM solution in DMSO-d_6 or CD_3CN respectively) for 1 h at room temperature to yield coordination cage $[Pd_2\mathbf{L}_4](BF_4)_4$ quantitatively.

^{1}H NMR (500 MHz, DMSO-d_6): δ [ppm] = 9.26 (b, H_g, and H_f, 4H), 8.45 (s, H_a, 2H), 8.34 (d, J = 7.8 Hz, H_e, 2H), 7.85 (d, J = 7.5 Hz, H_e, 2H), 7.78 (dd, J = 8.5 Hz, H_b, 2H), 7.72 (d, J = 7.9 Hz, H_c, 2H), 5.12 (b, H_o,1H), 4.58 (b, H_h, 2H), 4.08 (b, H_n,1H), 3.93 (b, H_m, 2H), 3.75 (b, H_i, 2H), 3.41 (b, H_l, 2H), 3.33-3.32 (b, H_j, and H_k, 4H), 2.01–2.08 (b, 2H), 1.85–0.72 (m, 38H), 0.44 (s, 3H).

^{13}C NMR (150 MHz, DMSO-d_6): δ [ppm] = 153.5, 150.9, 149.5, 143.6, 141.4, 138.8, 131.5, 127.4, 123.4, 123.0, 122.2, 121.6, 111.5, 111.4, 97.8, 83.09, 76.9, 70.1, 69.7, 69.1, 68.1, 66.4, 55.9, 55.5, 49.1, 41.6, 37.5, 35.7, 35.2, 31.3, 31.1, 27.4, 27.1, 23.8, 23.4, 22.7, 22.4, 20.4, 18.5, 11.3

ESI-FTICR-HRMS $[(C_{60}H_{71}N_3O_5)_4Pd_2]^{4+}$: found: 967.2430; calc.: 967.2426

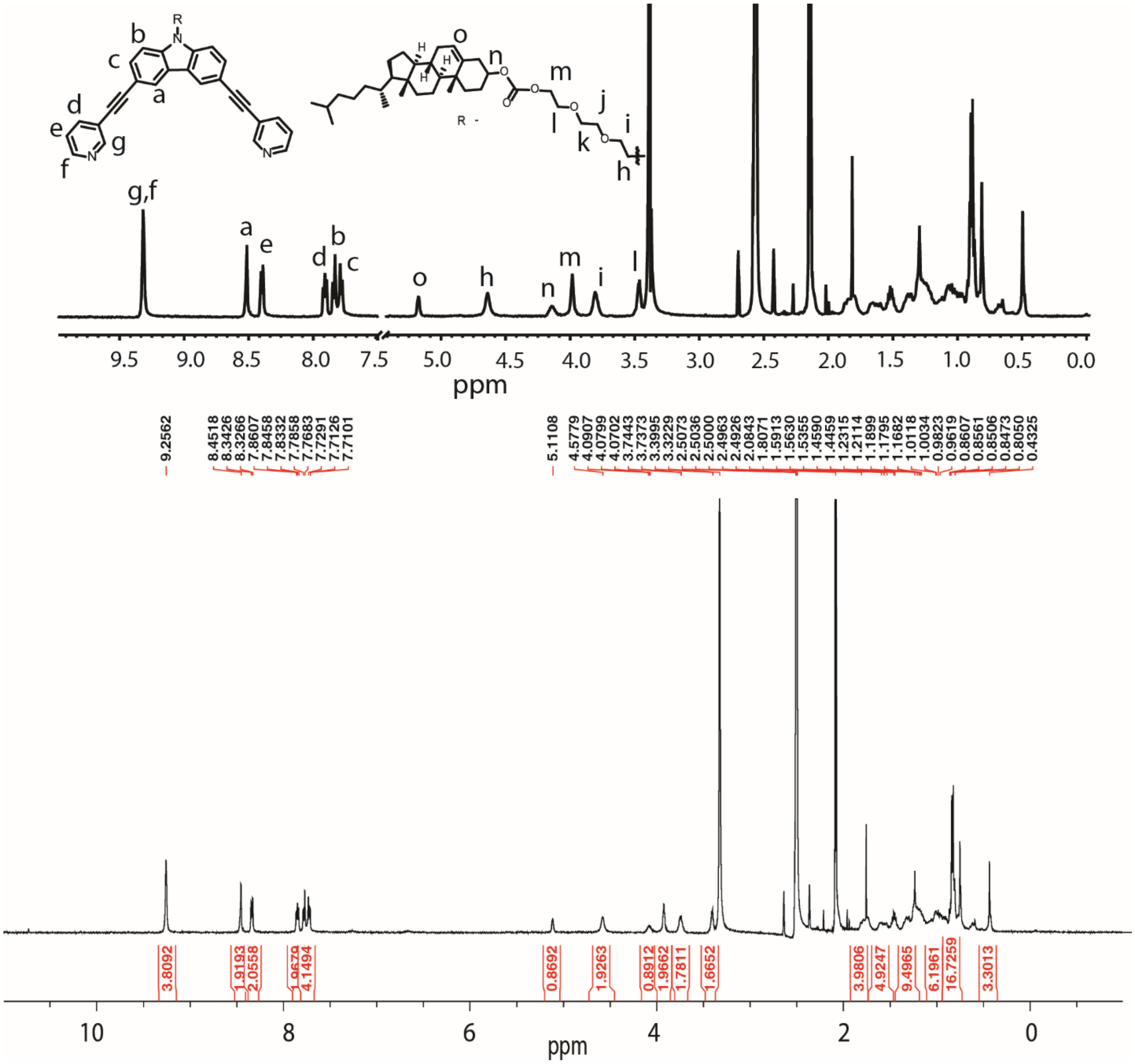

Figure S10: ^{1}H NMR spectrum (500 MHz, 298K, DMSO-d_6) of cage $[Pd_2\mathbf{L}_4](BF_4)_4$: Full spectrum (bottom), selected region (top).

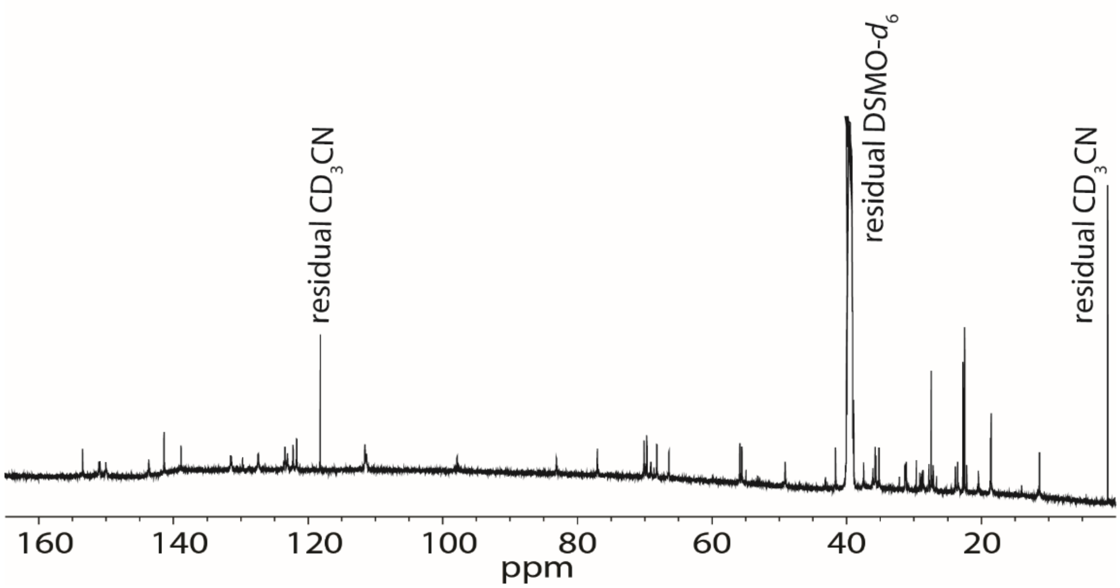

Figure S11: ^{13}C NMR spectrum (150 MHz, 298K, DMSO-d_6) of cage $[Pd_2\mathbf{L}_4](BF_4)_4$

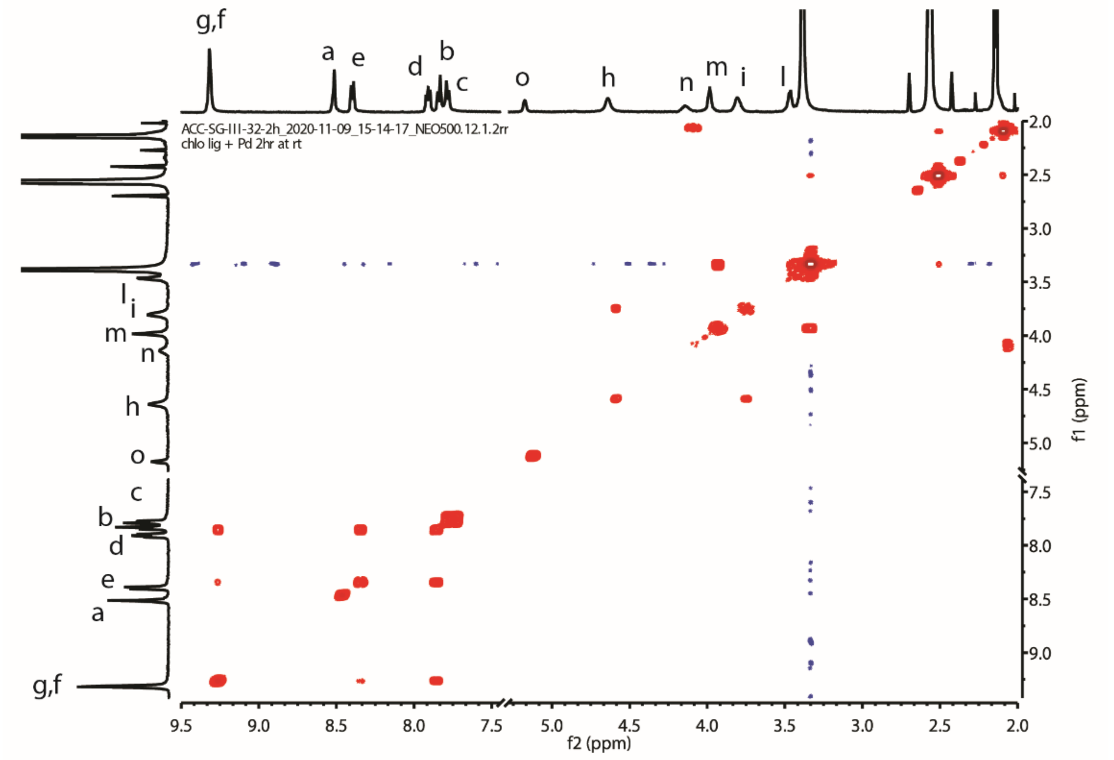

Figure S12: ^{1}H - ^{1}H COSY NMR spectrum (500 MHz, 298K, DMSO- d_6) of cage $[Pd_2\mathbf{L}_4](BF_4)_4$

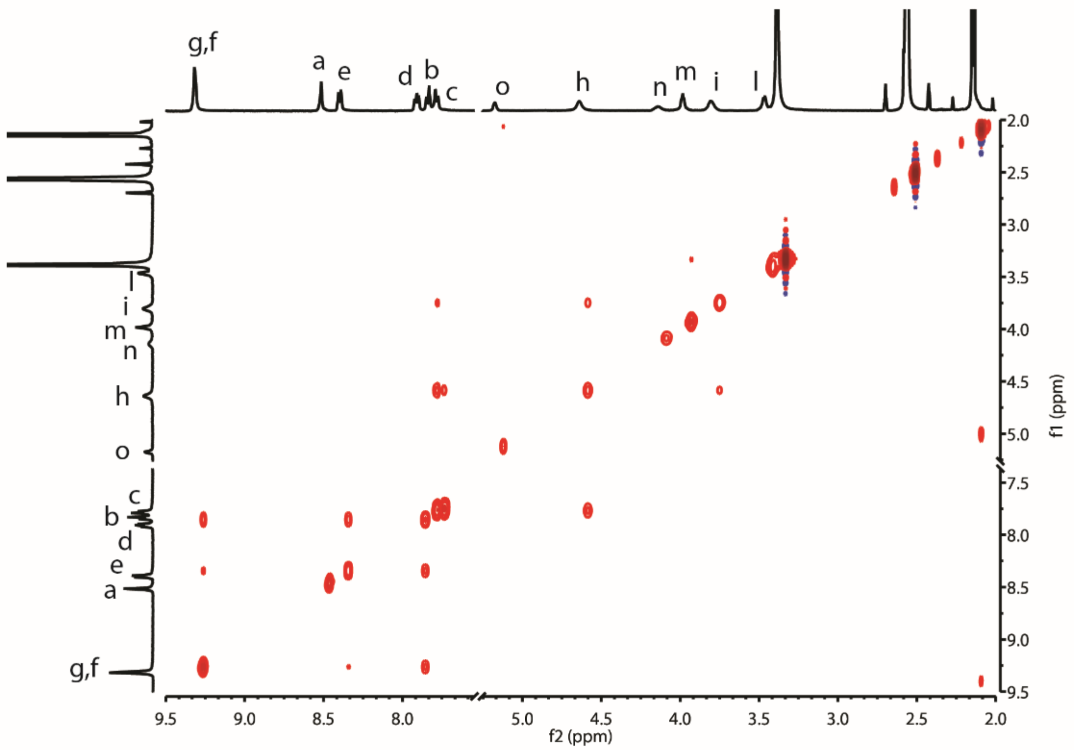

Figure S13: ^{1}H - ^{1}H NOESY NMR spectrum (500 MHz, 298K, DMSO-d_6) of cage $[Pd_2\mathbf{L}_4](BF_4)_4$

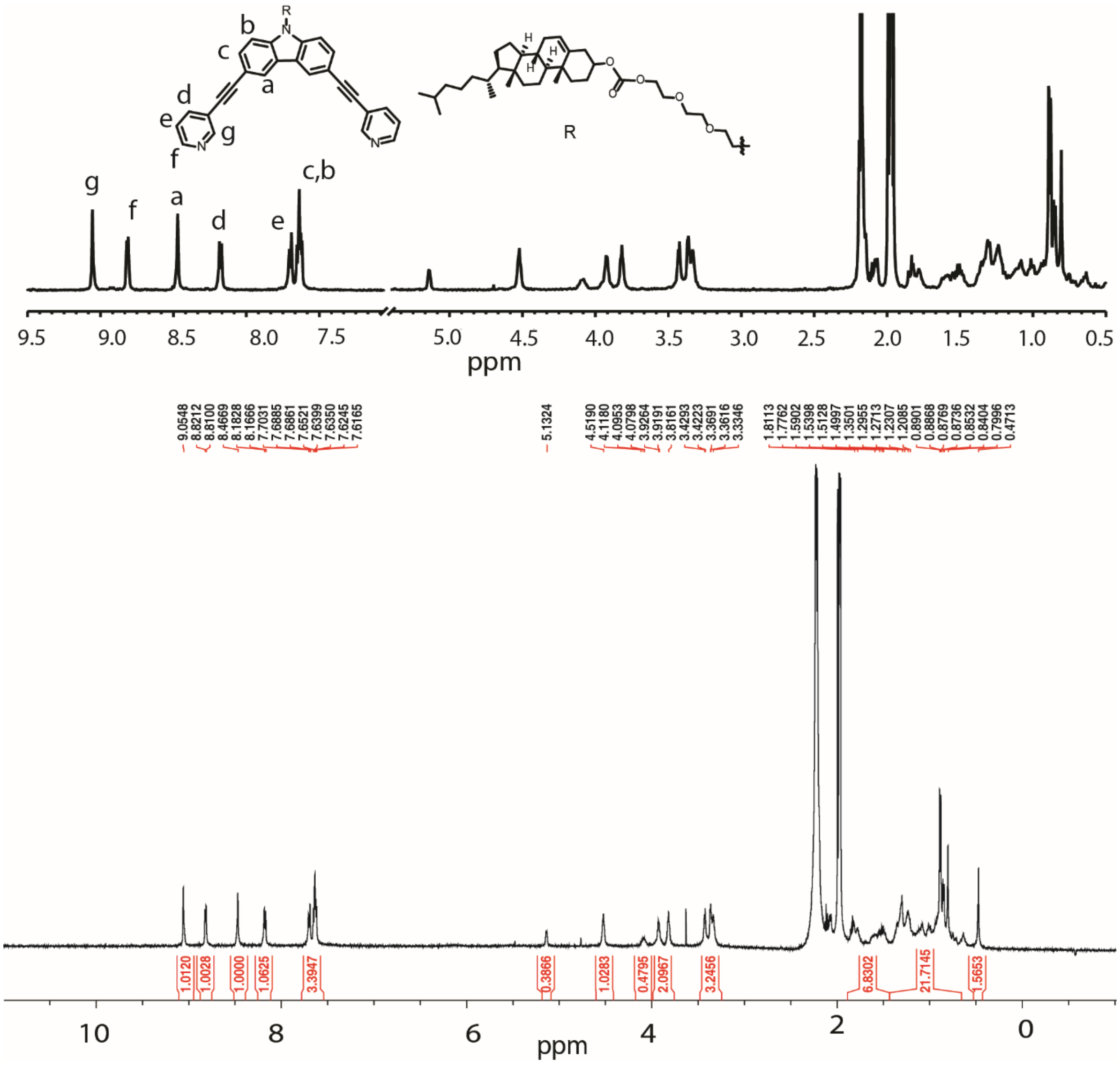

Figure S14: ^{1}H NMR spectrum (500 MHz, 298K, CD_3CN) of cage $[Pd_2\mathbf{L}_4](BF_4)_4$: Full spectrum (bottom), selected region (top).

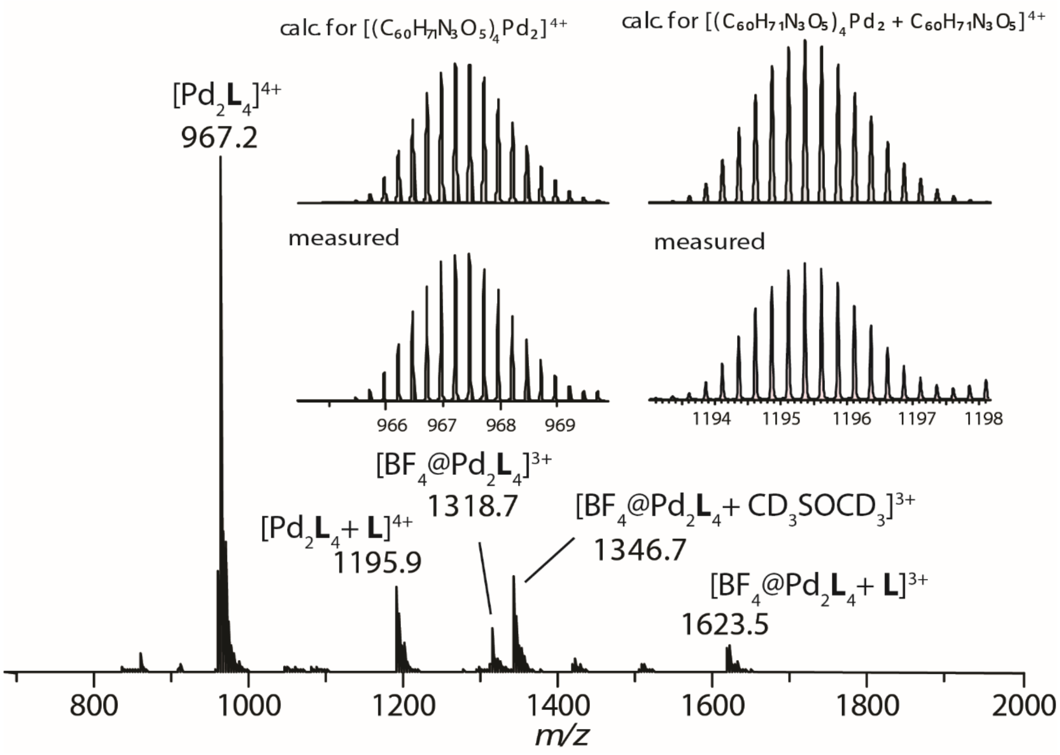

Figure S15: ESI-MS of the cage $[Pd_2\mathbf{L}_4](BF_4)_4$

3) ^{1}H-DOSY spectroscopy

^{1}H DOSY NMR spectra were recorded with a dstebpgp3s pulse sequence [1,2] with diffusion delays D20 of 0.06-0.10 s and gradient powers P30 of 800 to 2000 µs for each species optimized. Diffusion coefficients and hydrodynamic radius for ligand and cage were calculated with the Stokes-Einstein equation [3,4].

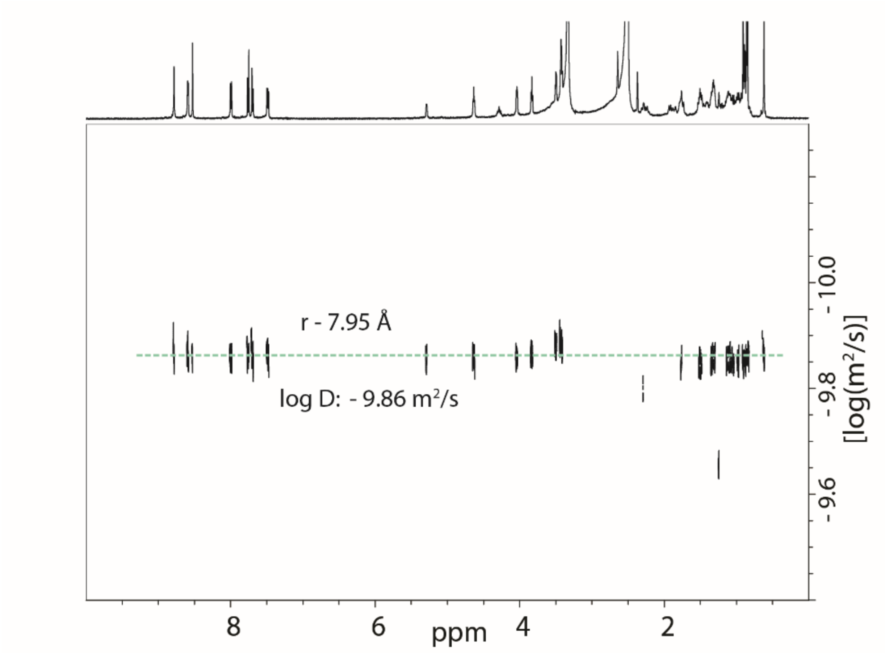

Figure S16: ^{1}H DOSY spectrum (500 MHz, 298K, DMSO-d_6) of ligand **L**.
Diffusion coefficient: 1.38×10^{-10} m^2s^{-1}, log D: -9.86, hydrodynamic radius = 7.95 Å.

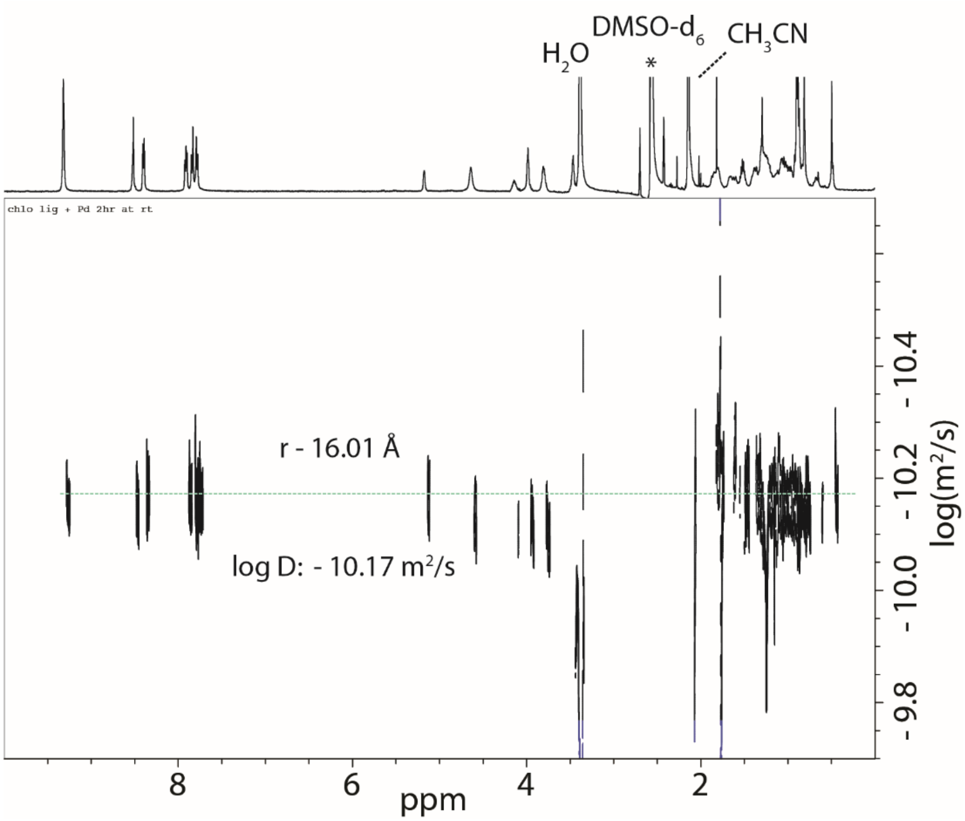

Figure S17: ^{1}H DOSY spectrum (500 MHz, 298K, DMSO-d_6) of cage $[Pd_2\mathbf{L}_4](BF_4)_4$. Diffusion coefficient: $6.817{\times}10^{-11}$ m^2s^{-1}, log D: -10.17, hydrodynamic radius = 16.09 Å.

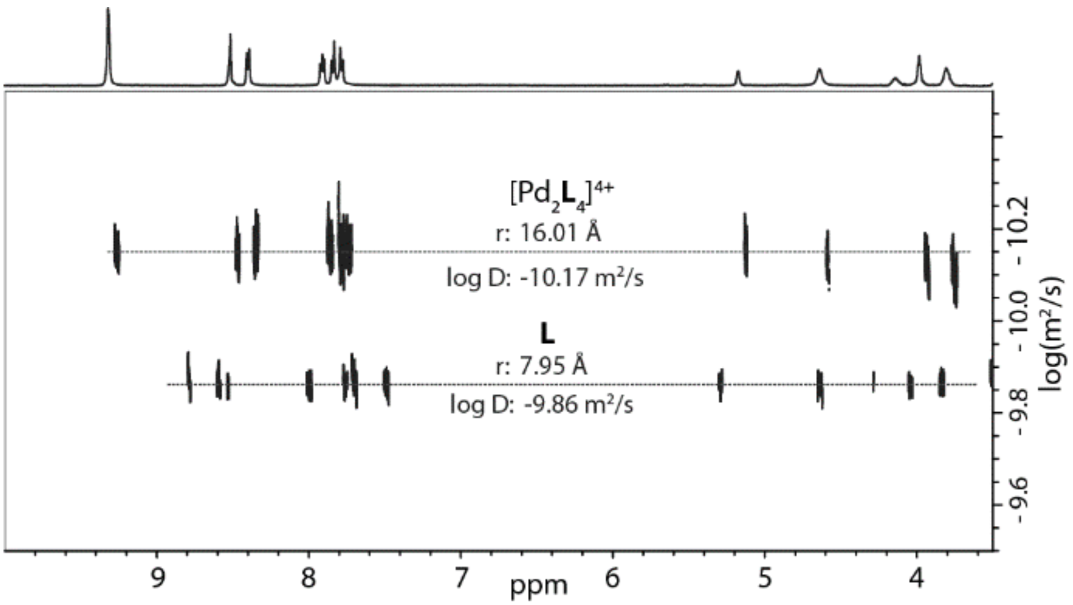

Figure S18: Comparison of ^{1}H DOSY spectra (500 MHz, 298K, DMSO-d_6) of ligand and cage (selected region).

4) Geometry optimization of cage $[Pd_2L_4]^{4+}$

Structural models of ligand and cage (Figure S19) were optimized on semiempiric PM6 level using the SPARTAN′18[5] software package without any constraints.

The structural models listed in the Figure S20 and Figure S21 were optimized using the semiempiric GFN2-xTB (using the xtb software) without any constraints.[6]

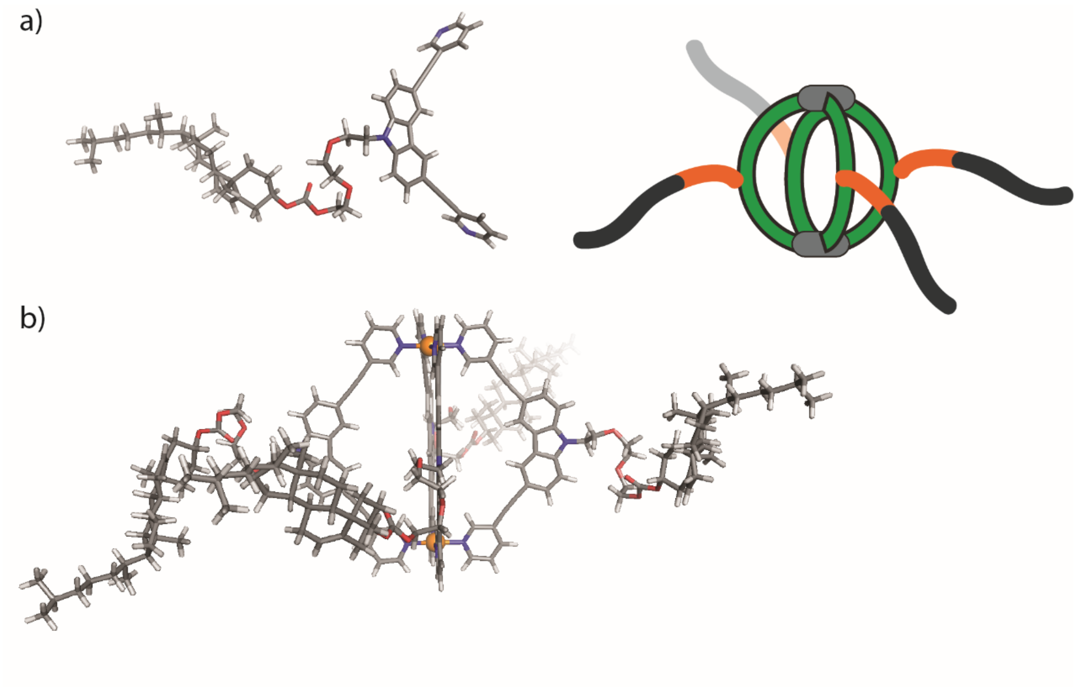

Figure S19: Geometry-optimized structures of ligand **L** and cage $[Pd_2\mathbf{L}_4]^{4+}$ (semiempiric PM6 method in SPARTAN′18 without any constraints).

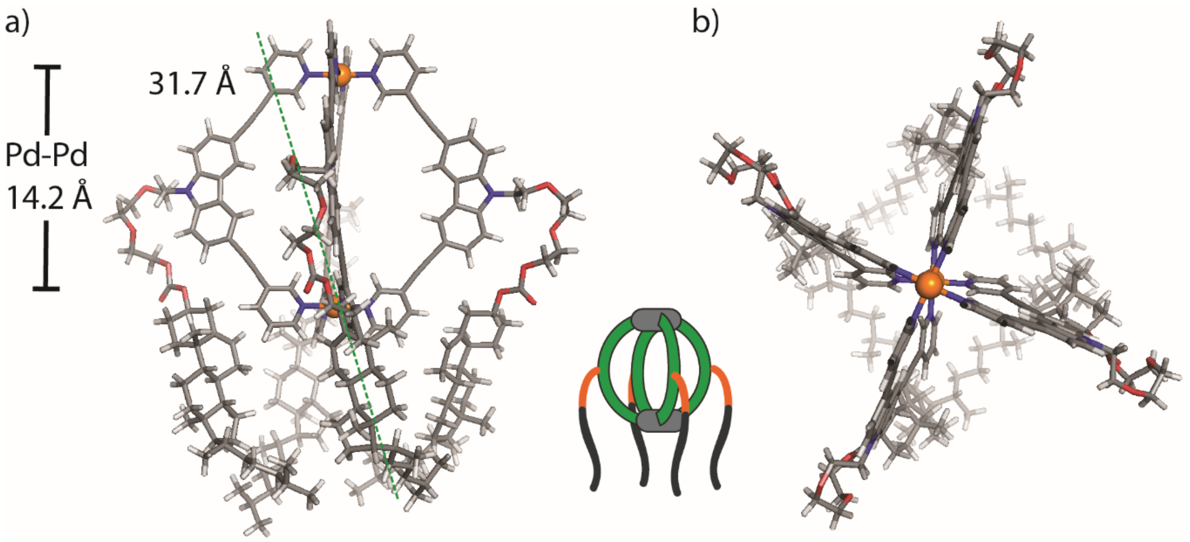

Figure S20: A structural model of the cage with cholesterol groups coming together vertically towards one of the metal sites (semiempiric GFN2-xTB without any constraints, using the xtb software). In this conformation, the distance between the Pd atoms is ~14 Å, which is similar to the reported cage framework.[7] A representative diagonal diameter (indicated in the figure) of the cage is about ~ 32 Å. The diameter obtained from the ^{1}H DOSY calculation is similar to this proposed structure.

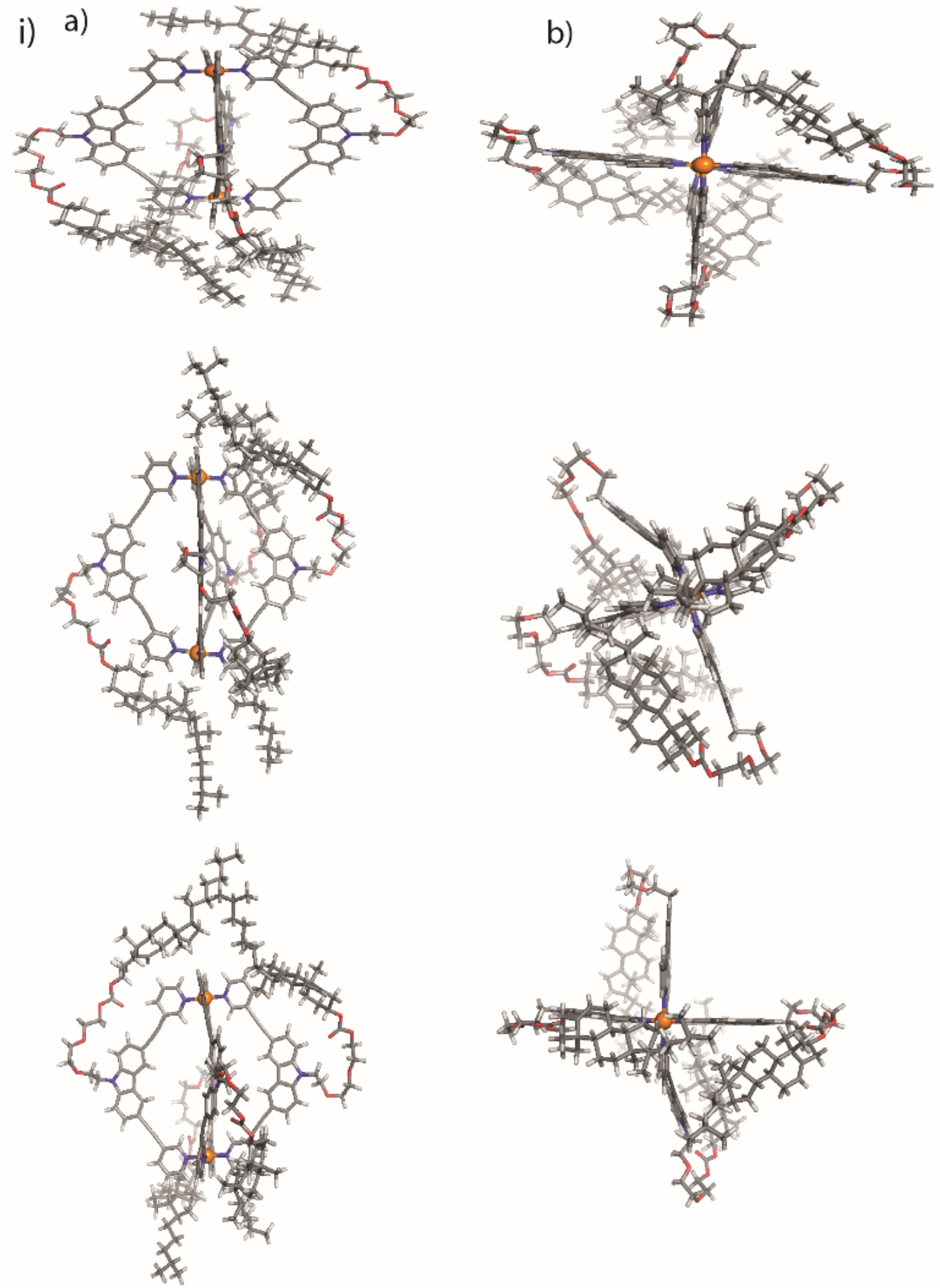
i)
a)
b)

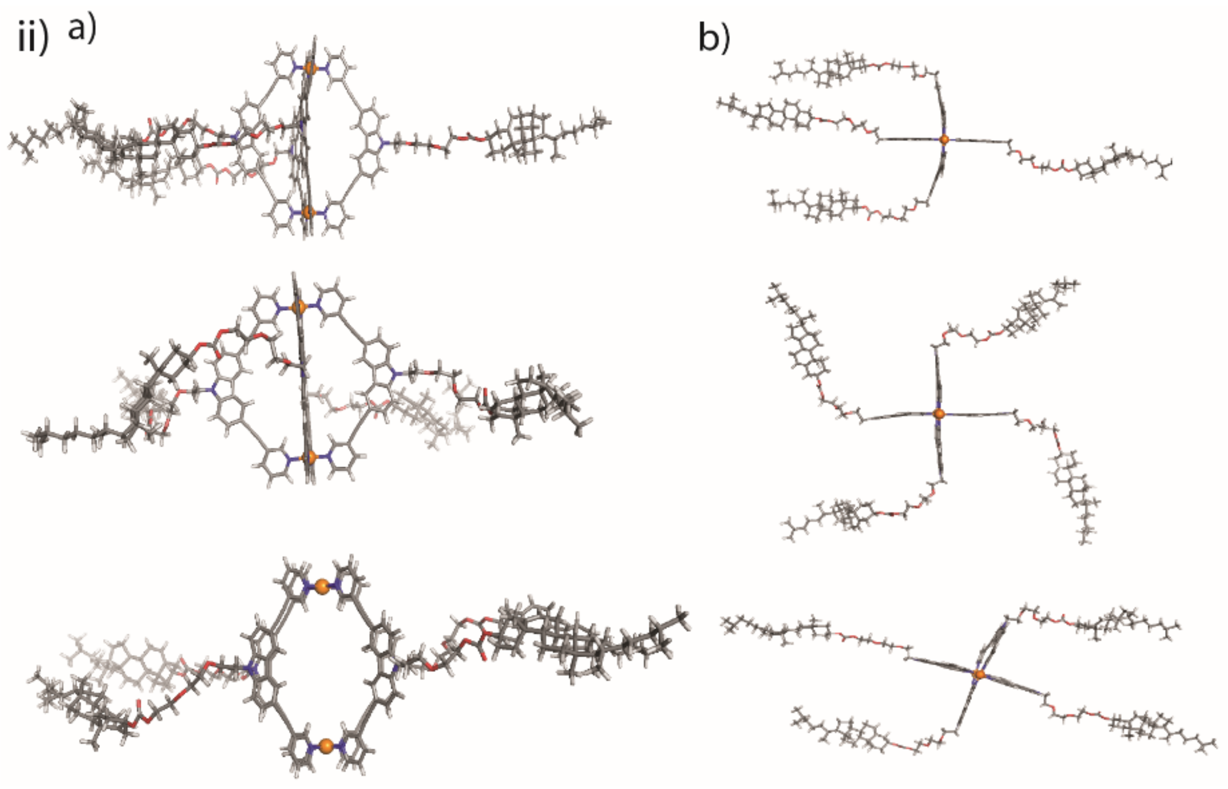

Figure S21: i) Structural models of the cage aligning cholesterol groups in different directions: i) cholesterol groups aligned towards metal centers, and ii) cholesterol groups aligned away from the cage. Views a) along the Pd-Pd axis (top view), and b) through the Pd-Pd axis (side view). To obtain these models, first, short (10 ps) MD simulations with multiple constraints were performed using the GFN-FF force field and the implicit solvent model GBSA. Then, the last structure of the trajectory was optimized with the semiempirical model GFN2-xTB and the implicit solvent model ALPB without any constraints.

This procedure yielded a collection of further structures that are worth comparing with the two initial models depicted in Figure S20 (all chains extended and all chains snug-fit associated with the cage core towards one Pd-face). In terms of morphological features that seem plausible for bestowing the compound with amphiphilic character, we further identified the first conformation shown in Figure S21.ii (pitchfork-like shape with three chains extending to one side and one to the opposite within the cage's equatorial plane) as alternative to the one shown in Fig. S. 20, as it has a much higher molecular dipole moment (130.3 D) than the latter structure (12.9 D), according to GFN2-xTB.

5) Hierarchical assembly of aggregates based on the amphiphilic cage

0.5 mL of bidestilled water (filtered through 0.25 µm syringe filter) was added to the cage solution (0.5 mL, 0.7 mM in CD_3CN, filtered through 0.25 µm syringe filter) and vortexed for 5 min. The freshly prepared sample was used to study the self-aggregation behaviour by transmission electron microscopy (TEM) and dynamic light scattering (DLS) methods.

5.1 DLS analysis of cage in MeCN: Water (1:1)

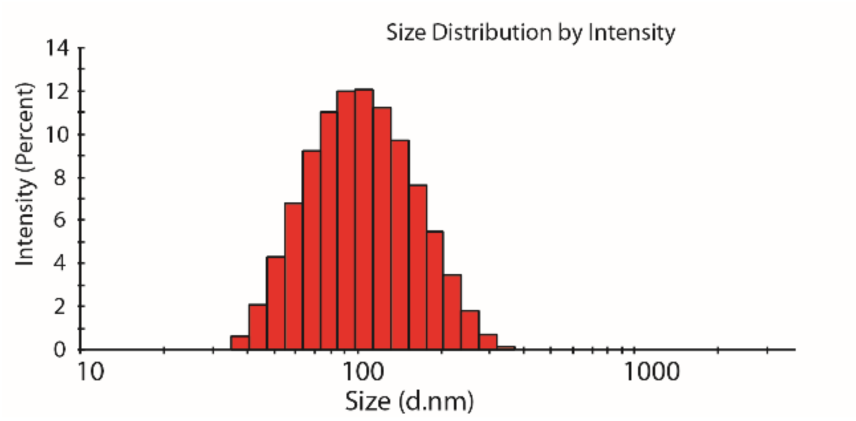

Figure S22: DLS analysis of the cage (0.35 mM) in MeCN: water (1:1).

5.2 TEM analysis of cage in MeCN: Water (1:1)

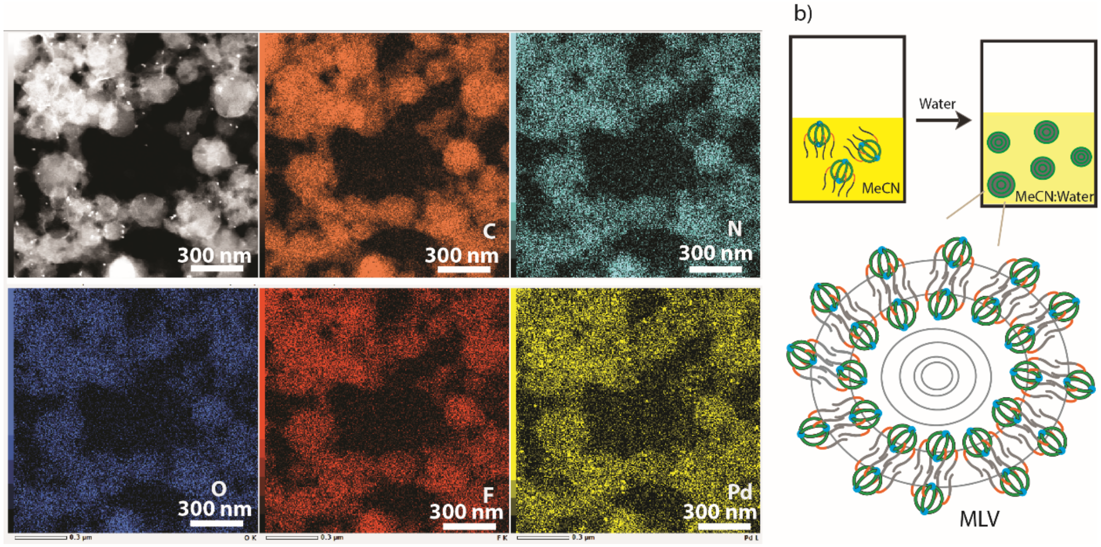

Figure S23: a) STEM analysis of the cage (0.35 mM) in MeCN: water (1:1) with energy dispersive X ray spectroscopy (EDS) analysis [scale bar: 300 nm; elements: C (orange), N (cyan), O (blue), F (red) and Pd (yellow)]; b) Schematic representation of multilamellar vesicle formation of the cage in MeCN : water (1:1).

6) Foam formation

Experiments were done with solutions of ligand (6 mM) and cage (1.4 mM) in DMSO. After shaking the vials for 5 min, the samples were kept aside, and the foam formation and decay were observed. The vial containing only the ligand solution does not form any foam, while the vial with the cage solution forms a foam (air in oil type emulsion), immediately. The foam is stable for 8 h without much change in volume.

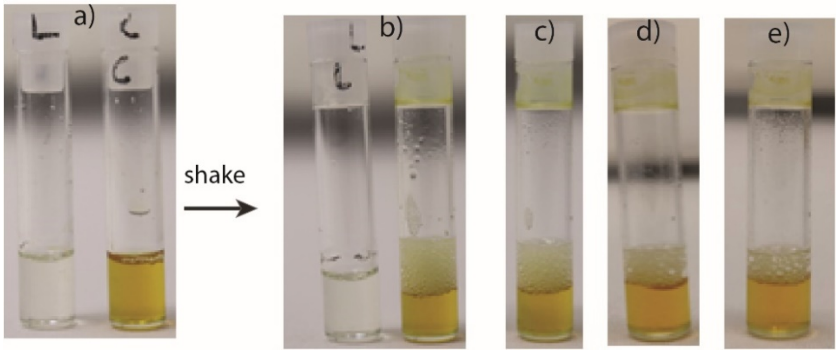

Figure S24: Foam formation and stability studies: Ligand (6 mM) and cage (1.4 mM) in DMSO a) before and b) after shaking for 5 min; stability of the foam after c) 8 h, d) 24 h and e) 48 h without any external disturbance.

7) Oil-in-oil emulsification

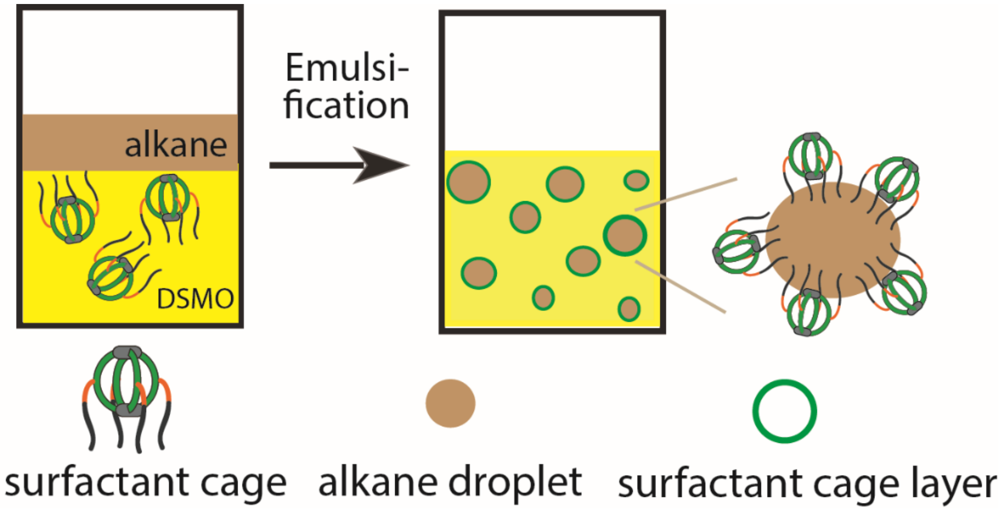

Figure S25: Schematic representation of emulsion formation

7.1 Method

Emulsions were prepared by 400 mg of continuous phase, i.e. DMSO (containing 0.5 mg cage) and 100 mg of dispersed phase, i.e. hexadecane (HD), *n*-octane (Oct), isopropyl palmitate (IPP), hexane (Hex) or cyclohexane (CH) (in the ratio of 4:1). In one example, a mixture of 400 mg of DMSO (containing 0.5 mg cage) and 400 mg of hexadecane (in the ratio of 1:1) was prepared. The mixtures were subjected to homogenization by vortexing at 3000 rpm for 10 minutes.

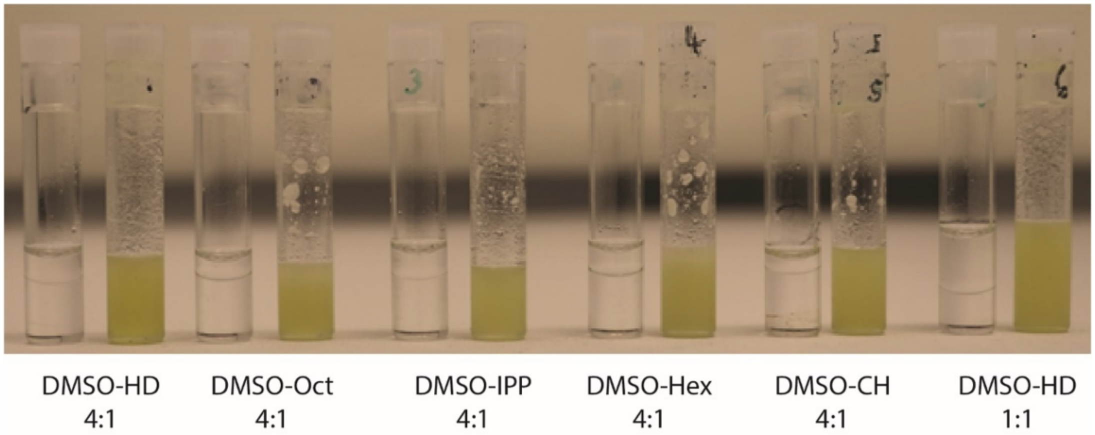

Figure S26. Images of emulsions DMSO: alkane = 4:1 and 1:1 without (left) and with (right) surfactant cage (0.1% w/w).

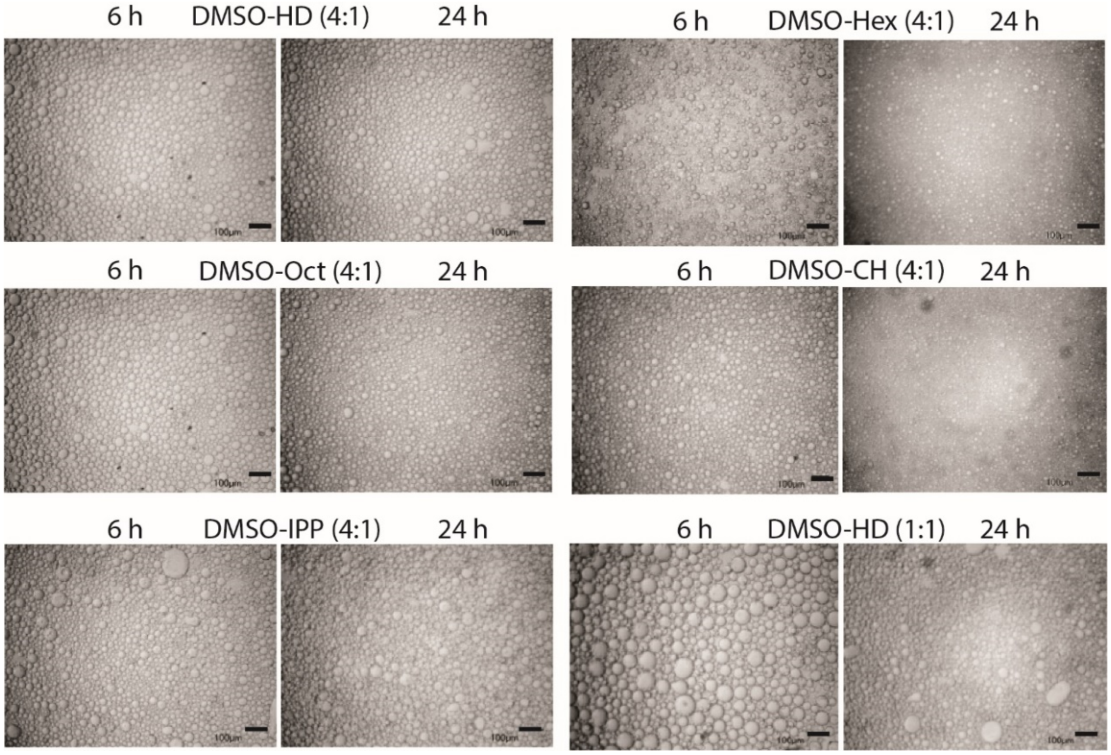

Figure S27: Optical microscopy images of the emulsions (DMSO: alkane = 4:1 and 1:1 with 0.1% cage, w/w) after 6 h and 24 h. Scale bar 100 µm.

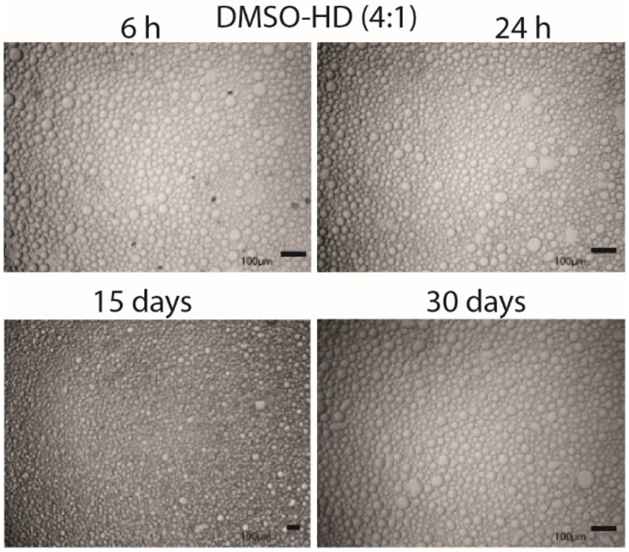

Figure S28: Time-dependent stability: Optical microscopy images of DMSO: HD emulsion (4:1 with 0.1% cage) after 6 h, 1 day, 15 days, and 1 month. Scale bar 100 µm.

8) Polymerization

8.1 Synthesis of polyurea nanoparticles

200 mg of hexadecane (dispersed phase) containing isocyanate monomer [(hexamethylene diisocyanate (HDI), 9.4 mg, 0.056 mmol) or (diphenylmethane-4,4'-diisocyanate (4,4′-MDI), 14.0 mg, 0.056 mmol)], was introduced into 750 mg of DMSO (continuous phase) containing 2 mg of surfactant cage (0.2% w/w) and vortexed at 3000 rpm for 10 min to make an emulsion with polymer monomer in hexadecane droplets. The other polymer monomer 1,4 diaminobutane (4.9 mg in 50 µL DMSO, 0.056 mmol), was added to the emulsion and then stirred for 60 min. Then 1 mL water was added, and the suspension was filtered in a centrifugal Eppendorf vial with filter inset and the solids were collected. Further, the solids were characterized by TEM methods and FT-IR spectroscopy.

8.2 Synthesis of polyurethane nanoparticles

200 mg of hexadecane containing isocyanate monomer [(HDI, 9.4 mg, 0.056 mmol) or (4,4′-MDI, 14.0 mg, 0.056 mmol)], and dibutyltin dilaurate catalyst (DBTDL, 3.5 mg, 0.0056 mmol) was introduced into 750 mg of DMSO (continuous phase) containing 2 mg of surfactant cage (0.2% w/w emulsifier) and vortexed at 3000 rpm for 5-10 min to make an emulsion with polymer monomer in hexadecane droplets. The other polymer monomer 1,4 butanediol (5 mg in 50 µL DMSO, 0.056 mmol) was introduced into the emulsion then stirred for 15 min. Then temperature was raised to 60 °C, and continued stirring for 4 h. Then 1 mL water was added, and the suspension was filtered in a centrifugal Eppendorf vial with filter inset and the solids were collected. Further, the solids were characterized by TEM methods and FT-IR spectroscopy.

The mean size of polymer particles (~1.00 ± 0.25 µm, and ~2.00 ± 0.30 µm for 4,4'-MDI and HDI PUs respectively, and ~45 ± 11 nm, and ~85 ± 25 nm for 4,4'-MDI and HDI PUTs, respectively) was extracted from TEM experiments. Polymer particles produced from the cage-like surfactant derived emulsions are more or less comparable to other polymer products prepared using oil-in-water or oil-in-oil emulsions.[8-11]

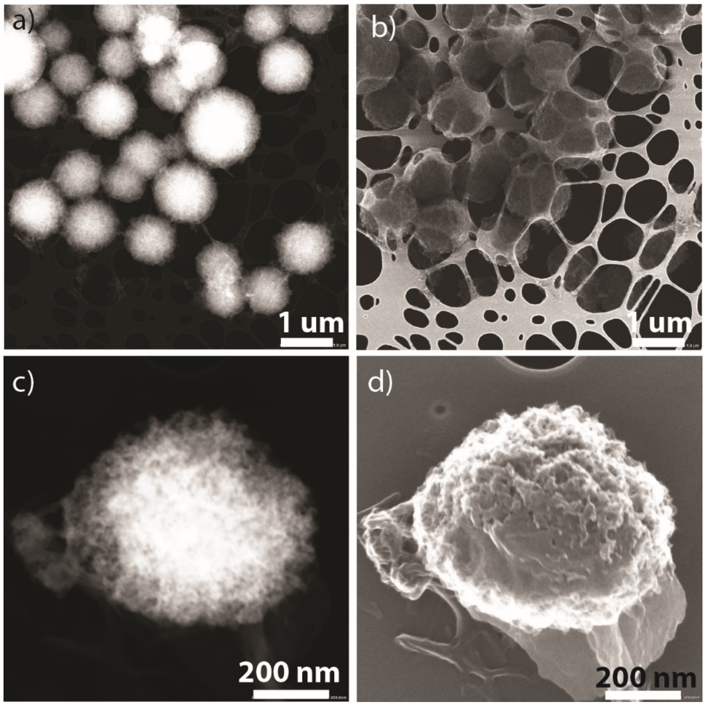

Figure S29. Polyurea nanoparticles of 4,4′-MDI -polyurea visualized scanning transmission electron microscopy–high-angle annular dark-field microscopy (STEM-HAADF) (a and c) and scanning electron microscopy (SEM) (b and d). Individual spherical particles are shown in c) and d).

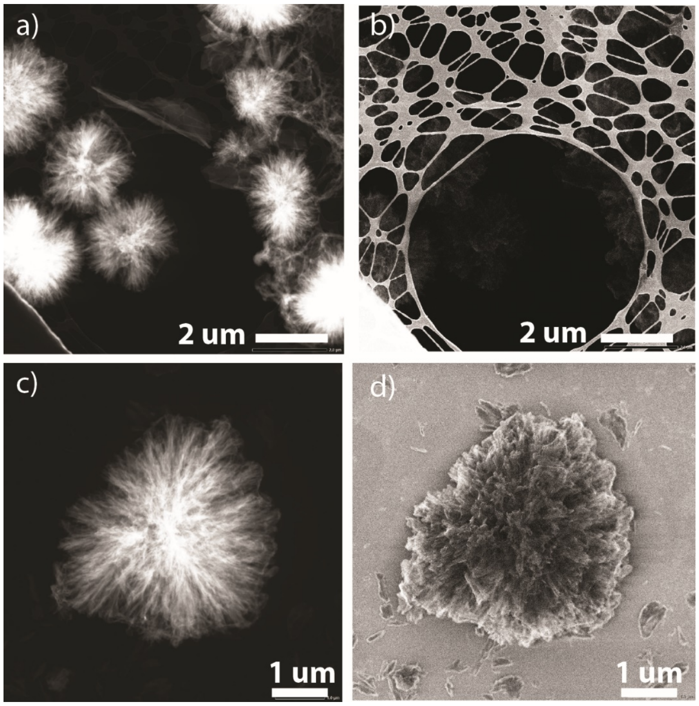

Figure S30. Polyurea nanoparticles of HDI-polyurea visualized by STEM-HAADF (a and c) and SEM (b and d). Individual cotton wool kind of particles are shown in c) and d).

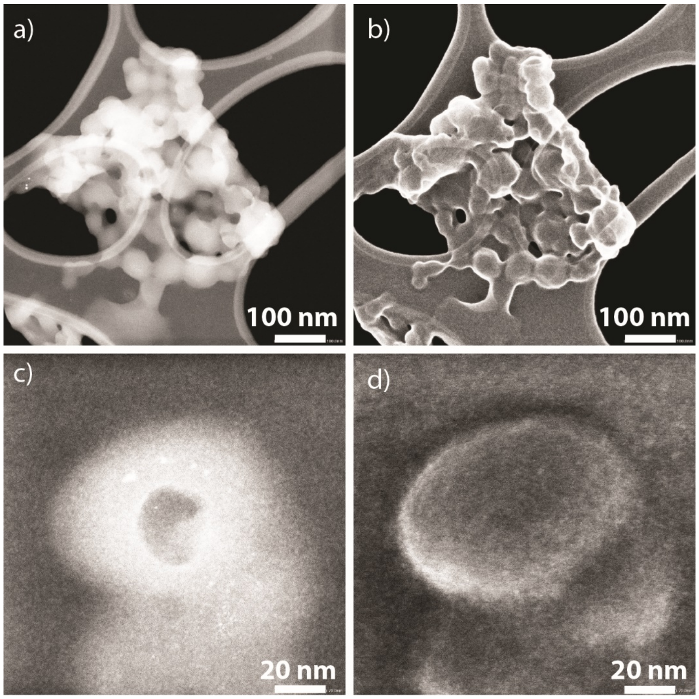

Figure S31. Polyurethane nanoparticles of 4,4′-MDI -polyurethane visualized by STEM-HAADF (a and c) and SEM (b and d). Individual pearl-shaped particles are shown in c) and d).

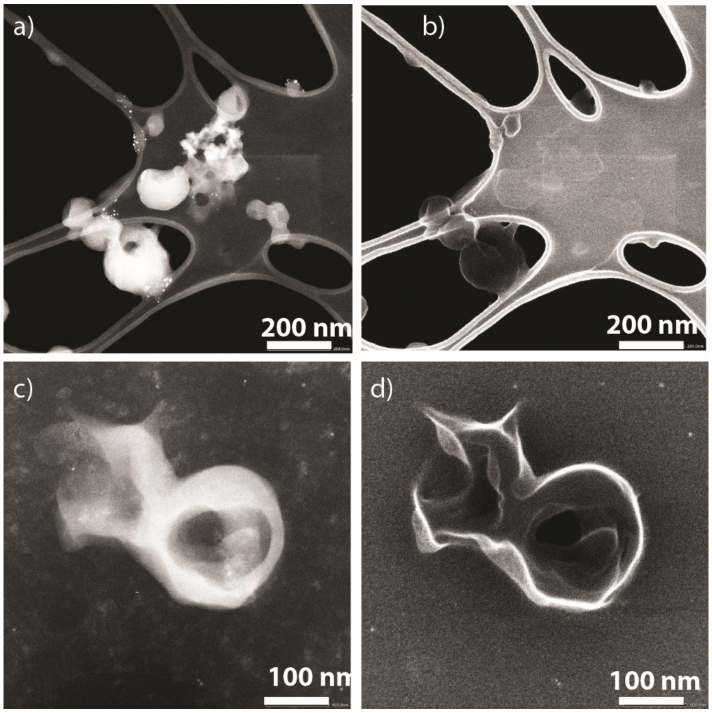

Figure S32. Polyurethane nanoparticles of HDI-polyurethane visualized by STEM-HAADF (a and c) and SEM (b and d). Individual pearl-shaped particles are shown in c) and d).

8.3 FT-IR spectra of polyurethane particles

FT-IR spectra of polyurethane and polyurea particles were depicted in figures S33 and S34. The absence of an absorption band at the characteristic of N=C=O group stretching vibration (2259 for cm^{-1} 4,4'-MDI and 2240 cm^{-1} for HDI) and the appearance of the bands in the region of 1700-1600 cm^{-1} (characteristic of C=O group stretching vibrations in urethane and urea) confirms the presence of urea or urethane in the polymer particles. The FT-IR spectra of polyurea particles (3317, 2914, 1632, 1554, and 1223 cm^{-1} for 4,4'-MDI-polyurea; 3331, 2929, 1618, 1553 and 1229 cm^{-1} for HDI-polyurea), and polyurethane particles (3309, 2934, 1689, 1526, 1250, and 1221 cm^{-1} for 4,4'-MDI polyurethanes; 3334, 2923, 1680, 1538, 1254, and 1221 cm^{-1} for HDI polyurethanes) are comparable to the literature reports.[11-14]

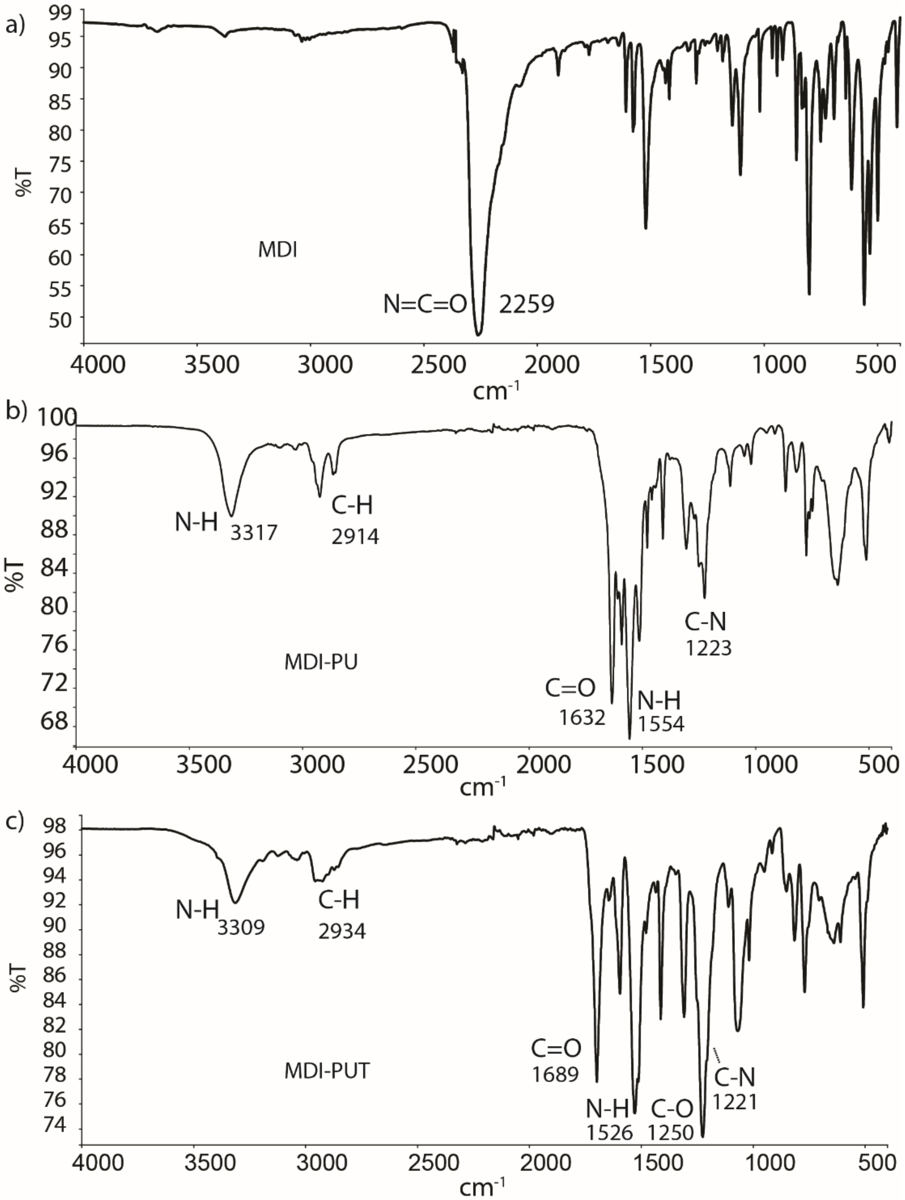

Figure S33. FT-IR spectra of a) 4,4′-MDI, b) polyurea made of 4,4′-MDI, and c) polyurethane made of 4,4′-MDI

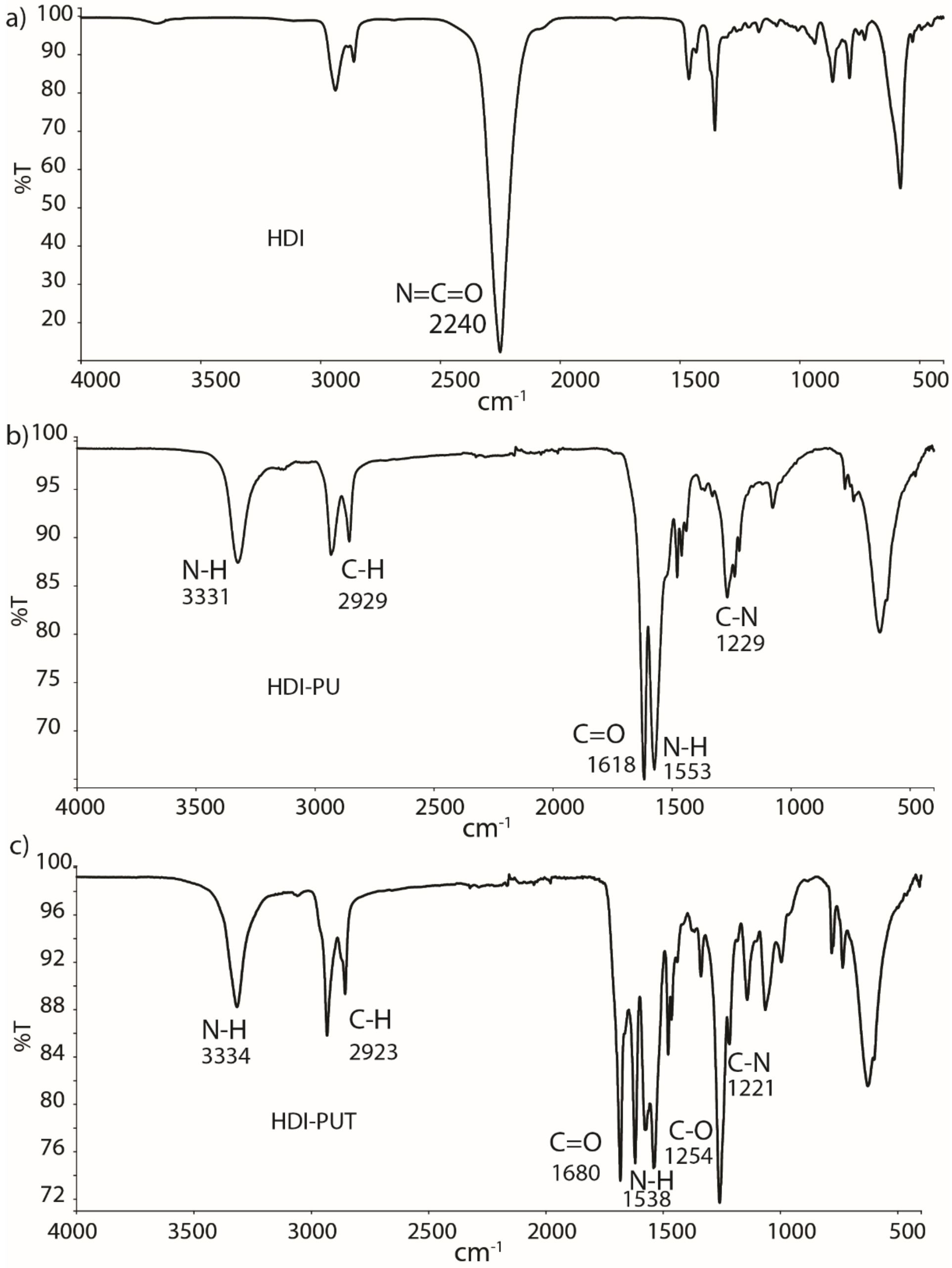

Figure S34. FT-IR spectra of a) HDI, b) polyurea made of HDI, and c) polyurethane made of HDI

9) Host-guest chemistry of the amphiphilic cage

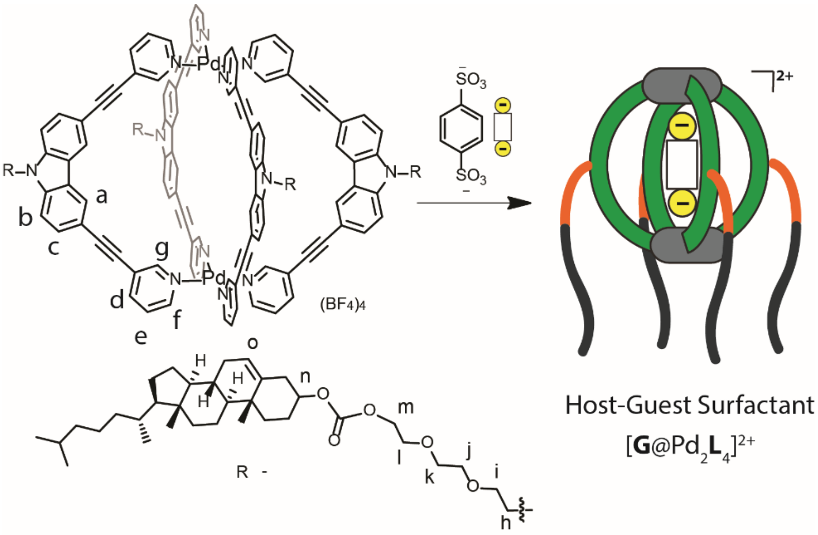

Scheme S2. Supramolecular host-guest assembly of [**G**@Pd_2**L**$_4$]$^{2+}$.

9.1 ^{1}H NMR spectroscopy studies of the host guest assembly: titration of G to [Pd_2L_4]$^{4+}$cage

The guest uptake by the amphiphilic cage was studied by titrating a solution of the benzene 1,4- di sulphonate (n-butyl ammonium salt, in DMSO-*d*$_6$, 10 mM) in step by step into 500µL of a 0.7 mM solution of the cages in DMSO-*d*$_6$ in an NMR tube.

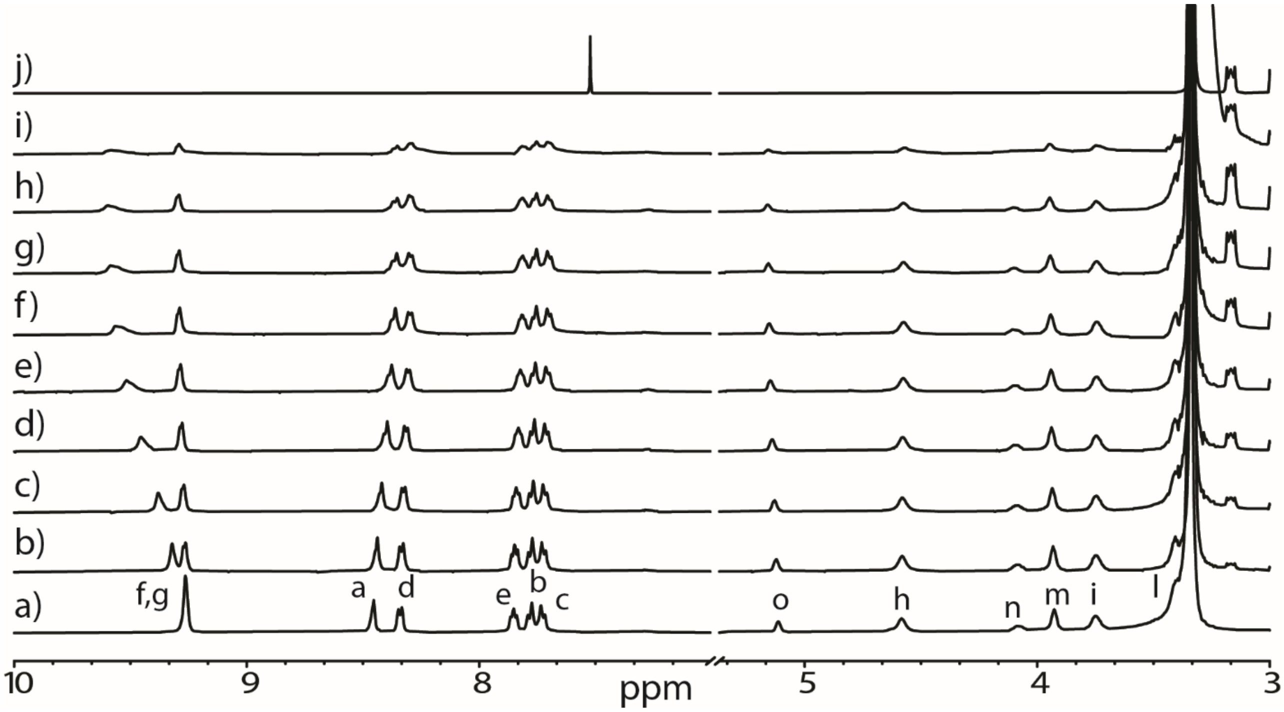

Figure S35: **G** titration experiment: ^{1}H NMR (500 MHz, DMSO-*d*$_6$, 0.7 mM) of a) [Pd_2**L**$_4$](BF_4)$_4$, after addition of b) 0.10 equiv., c) 0.20 equiv., d) 0.30 equiv., e) 0.40 equiv., f) 0.50 equiv., g) 0.70 equiv., h) 0.90 equiv., i) 1.0 equiv., of **G** (10 mM solution in DMSO-*d*$_6$) with respect to [Pd_2**L**$_4$](BF_4)$_8$, and j) free **G**.

9.2 ESI-MS of the $[G@Pd_2L_4]^{2+}$

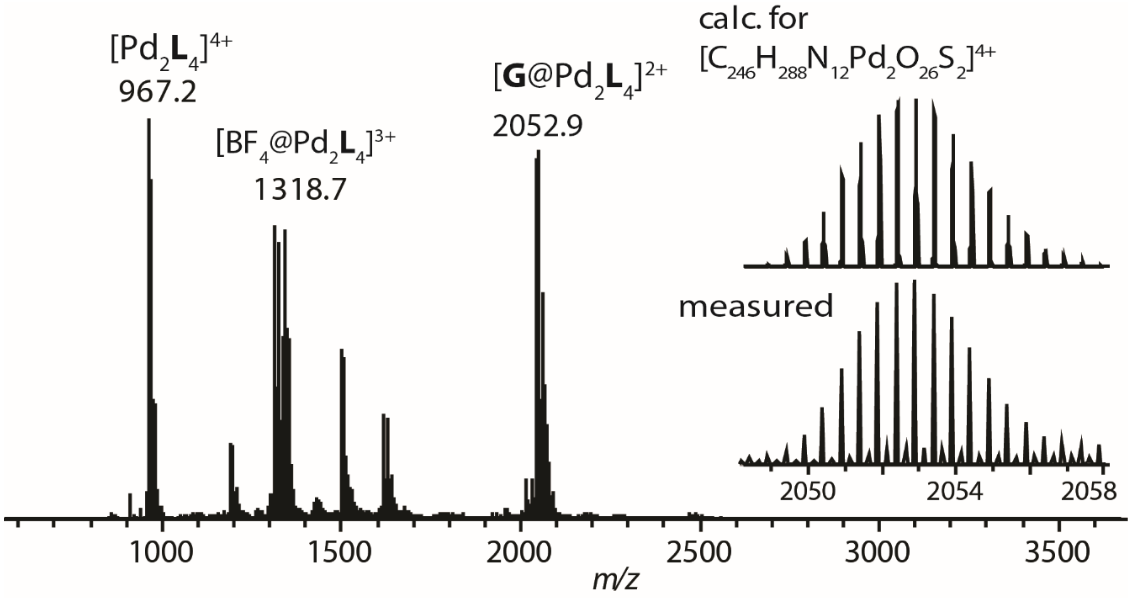

Figure S36: ESI-MS of the cage $[Pd_2\mathbf{L}_4](BF_4)_4$.

9.3 Oil-in-oil emulsification by host-guest adduct $[G@Pd_2L_4]^{2+}$.

Emulsions were prepared by 400 mg of DMSO (containing 0.5 mg $[\mathbf{G}@Pd_2\mathbf{L}_4]^{2+}$) and 100 mg of hexadecane (HD) in the ratio of 4:1. The mixtures were subjected to homogenization by vortexing at 3000 rpm for 10 minutes to obtain a nonaqueous emulsion.

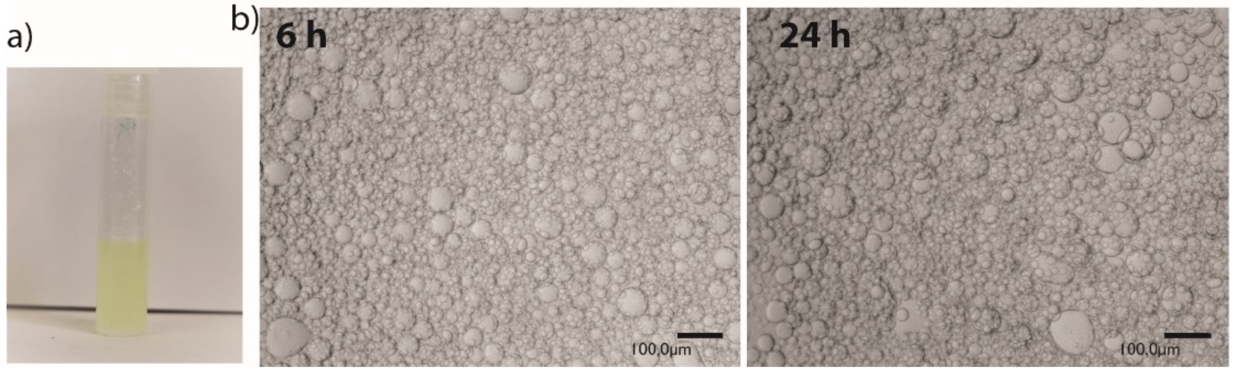

Figure S37. Image of emulsion (DMSO: HD = 4:1) with $[\mathbf{G}@Pd_2\mathbf{L}_4]^{2+}$ surfactant (0.1% w/w). Optical microscopy images of the emulsion after 6 h and 24 h. Scale bar 100 µm.

9.4 Synthesis of polyurea nanoparticles by employing $[G@Pd_2L_4]^{2+}$ as surfactant.

200 mg of hexadecane (dispersed phase) containing isocyanate monomer [hexamethylene diisocyanate (HDI), 9.4 mg, 0.056 mmol) or diphenylmethane-4,4'-diisocyanate (4,4′-MDI), 14.0 mg, 0.056 mmol)], was introduced into 750 mg of DMSO (continuous phase) containing 2 mg of surfactant cage (0.2% w/w) and vortexed at 3000 rpm for 10 min to make an emulsion with polymer monomer in hexadecane droplets. The other polymer monomer 1,4 diaminobutane (4.9 mg in 50 µL DMSO, 0.056 mmol), was added to the emulsion and then stirred for 60 min. Then 1 mL water was added, and the suspension was filtered in a centrifugal Eppendorf vial with filter inset and the solids were collected. Further, the solids were characterized by TEM methods.

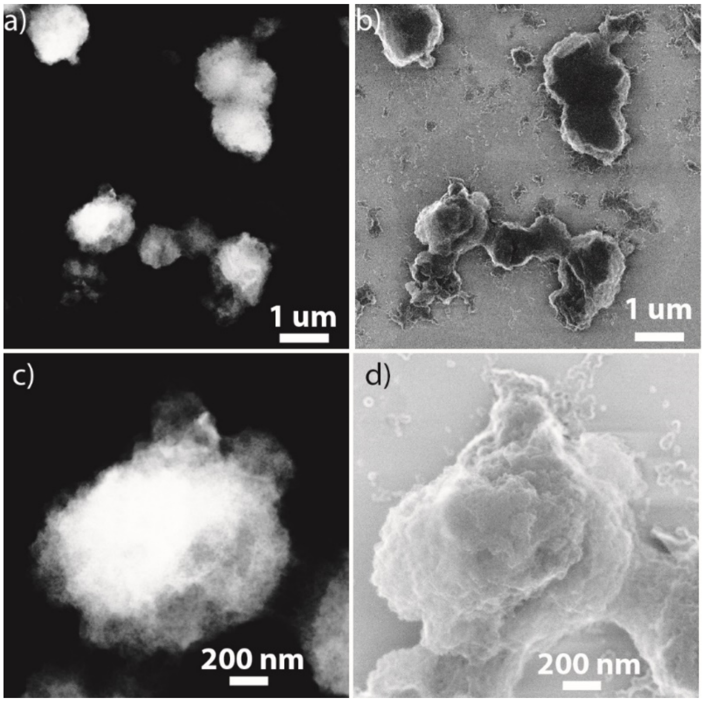

Figure S38. Polyurea nanoparticles of 4,4′-MDI -polyurea obtained from host-guest adduct surfactant visualized scanning transmission electron microscopy–high-angle annular dark-field microscopy (STEM-HAADF) (a and c) and scanning electron microscopy (SEM) (b and d). Individual spherical particles are shown in c) and d).

10) References

[1] A. Jerschow, N. Müller, *J. Magn. Reson.* **1996**, *123*, 222–225.

[2] A. Jerschow, N. Müller, *J. Magn. Reson.* **1998**, *132*, 13–18.

[3] A. Macchioni, G. Ciancaleoni, C. Zuccaccia, D. Zuccaccia, *Chem. Soc. Rev.* **2007**, *37*, 479–489.

[4] L. Avram, Y. Cohen, *Chem. Soc. Rev.* **2014**, *44*, 586–602.

[5] Spartan`18 Parallel Suite, Wavefunction, Inc., Irvine.

[6] a) C. Bannwarth, S. Ehlert, S. Grimme, *J. Chem. Theory Comput.* **2019**, *15*, 1652–1671; b) S. Spicher, S. Grimme, *Angew. Chem. Int. Ed.* **2020**, *59*, 15665–15673

[7] R. Zhu, J.Lübben, B. Dittrich, G. H. Clever, *Angew. Chem. Int. Ed.* **2015**, *54*, 2796–2800.

[8] M. Kobašlija, D. T. McQuade, *Macromolecules* **2006**, *39*, 6371–6375.

[9] M. Barrère, K. Landfester, *Macromolecules* **2003**, *36*, 5119–5125.

[10] D. Crespy, M. Stark, C. Hoffmann-Richter, U. Ziener, K. Landfester, *Macromolecules* **2007**, *40*, 3122–3135.

[11] F.Tiarks, K. Landfester, M. Antonietti, *J. Polym. Sci., Part A: Polym. chem.* **2001**, *39*, 2520– 2524

[12] H. Souguir, F. Salaün, P. Douillet, I. Vroman, S. Chatterjee, *Chemical Engineering Journal*, **2013**, *221*, 133-145.

[13] X. Chen, X. Liu, J. Lei, L. Xu, Z. Zhao, F. Kausar, X. Xie, X. Zhu, Y. Zhang and W. Z. Yuan, *Mol. Syst. Des. Eng.*, 2018, **3**, 364-375
